# Olympiad-level formal mathematical reasoning with reinforcement learning

**DOI:** 10.1038/s41586-025-09833-y

**Published:** 2025-11-12

**Authors:** Thomas Hubert, Rishi Mehta, Laurent Sartran, Miklós Z. Horváth, Goran Žužić, Eric Wieser, Aja Huang, Julian Schrittwieser, Yannick Schroecker, Hussain Masoom, Ottavia Bertolli, Tom Zahavy, Amol Mandhane, Jessica Yung, Iuliya Beloshapka, Borja Ibarz, Vivek Veeriah, Lei Yu, Oliver Nash, Paul Lezeau, Salvatore Mercuri, Calle Sönne, Bhavik Mehta, Alex Davies, Daniel Zheng, Fabian Pedregosa, Yin Li, Ingrid von Glehn, Mark Rowland, Samuel Albanie, Ameya Velingker, Simon Schmitt, Edward Lockhart, Edward Hughes, Henryk Michalewski, Nicolas Sonnerat, Demis Hassabis, Pushmeet Kohli, David Silver

**Affiliations:** Google DeepMind, London, UK

**Keywords:** Computer science, Computational science

## Abstract

A long-standing goal of artificial intelligence (AI) is to build systems capable of complex reasoning in vast domains, a task epitomized by mathematics with its boundless concepts and demand for rigorous proof. Recent AI systems, often reliant on human data, typically lack the formal verification necessary to guarantee correctness. By contrast, formal languages such as Lean^1^ offer an interactive environment that grounds reasoning, and reinforcement learning (RL) provides a mechanism for learning in such environments. Here we present AlphaProof, an AlphaZero-inspired^2^ agent that learns to find formal proofs through RL by training on millions of auto-formalized problems. For the most difficult problems, it uses test-time RL, a method of generating and learning from millions of related problem variants at inference time to enable deep, problem-specific adaptation. AlphaProof substantially improves state-of-the-art results on historical mathematics competition problems. At the 2024 International Mathematical Olympiad competition, our AI system, with AlphaProof as its core reasoning engine, solved three out of the five non-geometry problems, including the competition’s most difficult problem. Combined with AlphaGeometry 2^3^, this performance, achieved with multi-day computation, resulted in reaching a score equivalent to that of a silver medallist, marking the first time an AI system achieved any medal-level performance, to our knowledge. Our work demonstrates that learning at scale from grounded experience produces agents with complex mathematical reasoning strategies, paving the way for a reliable AI tool in complex mathematical problem solving.

## Abstract (web summary)

Olympiad-level formal mathematical reasoning is achieved with reinforcement learning, with performance reaching a score equivalent to that of a silver medallist at the 2024 International Mathematical Olympiad competition.

## Main

One of the grand challenges in artificial intelligence (AI) is to develop agents that can reason effectively and discover solutions in complex, open-ended environments. Mathematics, with its role as a foundation for scientific understanding, serves as a profound and meaningful domain in which to develop these capabilities. As a natural step towards this goal, we focus on developing the necessary reasoning capabilities within the domain of elite mathematics competitions. Although not open-ended themselves, these competitions are renowned for problems that demand a depth of creative and multi-step reasoning, thereby providing a crucial and standardized environment for measuring progress.

The historical arc of mathematics, from Euclid’s foundational axiomatization of geometry to the widespread adoption of symbolic algebraic notation, has been one of increasing formalization. Modern computer-verified systems such as the Lean proof assistant^1^ and collaborative libraries such as Mathlib^4^ represent the logical continuation of this trajectory, enabling the expression of complex mathematics in a machine-understandable format. In these systems, a formal proof is not just a sequence of arguments, but a specific data structure called a ‘proof term’ that encodes the entire logical argument from axioms to conclusion. Although these terms can be constructed directly, a user typically builds them interactively by applying actions called tactics: small programs that manipulate the current proof state—the set of hypotheses and goals—to advance the proof one logical step at a time. The soundness of this process is guaranteed by Lean’s kernel, which verifies that the generated proof term is a valid construction. These systems offer two transformative capabilities: first, the rigorous, automated verification of every logical step, guaranteeing proof correctness; and second, the transformation of mathematics into an interactive, verifiable domain, allowing mathematical reasoning to be treated as a process that can be simulated, experimented with and learned.

Reinforcement learning (RL) offers a powerful paradigm for learning through interaction and experience, where agents optimize their behaviour through trial and error to achieve specified goals. This approach has proven to be particularly adept at mastering complex domains where optimal strategies are unknown. The AlphaZero family of agents, for instance, demonstrated the ability to achieve superhuman performance in challenging board games such as Go, chess and shogi^2^, optimize quantum dynamics^5^, and discover more efficient algorithms for fundamental computations such as sorting^6^ and matrix multiplication^7^. The power of these systems stems from their ability to interact at scale with a verifiable environment and use grounded trial-and-error feedback to continually learn and refine their strategies. RL coupled with formal systems thus represents a particularly promising approach for tackling the challenge of automated mathematical reasoning.

Although formal systems provide verifiable grounding, considerable progress in AI mathematical reasoning has also occurred using large language models (LLMs) trained on vast corpora of informal, natural-language mathematical text. These models have shown impressive capabilities in solving a wide range of problems and generating human-like mathematical discourse^8–10^, benefiting directly from the scale and breadth of existing human knowledge expressed in text. Rigorously verifying the correctness of their reasoning remains, however, an active research challenge, currently using techniques such as checking final answers against known solutions or comparing, with systems that cannot be fully trusted, generated reasoning steps against reference proofs^11,12^. This lack of guaranteed correctness limits their reliability for validating mathematical claims or tackling problems without pre-existing reference points. In contrast, the inherent verification capabilities of formal systems provide the necessary foundation for building AI agents whose reasoning process and outputs can be trusted, even when exploring beyond the boundaries of existing human proofs and training data.

AlphaProof combines the rigour of formal systems with the experiential learning of RL to find proofs within the Lean theorem prover environment and to develop powerful mathematical reasoning. AlphaProof markedly improved state-of-the-art results on elite historical mathematics competition problems and, notably, proved three out of five problems at the 2024 International Mathematical Olympiad (IMO) competition. Although its solutions required computational time far exceeding that of human contestants, this success demonstrates the ability to tackle mathematical challenges previously considered beyond the reach of automated systems.

## AlphaProof

AlphaProof is an RL agent designed to discover formal mathematical proofs by interacting with a verifiable environment based on the Lean theorem prover. Its architecture, training and inference integrate several key innovations.

### The Lean RL environment

**Fig. 1:**
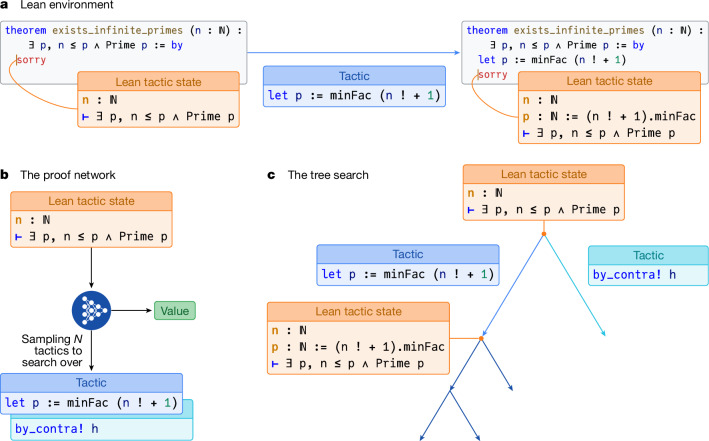
AlphaProof core reasoning components. **a**, The Lean environment in which each step of the proving process takes place. An agent observes an initial Lean tactic state (left), which describes the theorem to be proved. The ‘sorry’ keyword is a placeholder in Lean indicating that the proof is not yet complete. Applying a tactic to the environment (for example, ‘let p := minFac (n! + 1))’ results in a new state, potentially introducing new hypotheses or altering goals (right box). **b**, The proof network takes the current Lean tactic state as input and produces two outputs: a list of *N* promising tactics to try and a value estimating proof difficulty. **c**, The tree search uses the proof network’s outputs to guide its exploration of potential proof paths. Starting from an initial state (root node), the search iteratively expands the tree. At each node, it calls the proof network (**b**) to generate promising tactics and a value estimate for the current state. These tactics (edges) are then applied within the Lean environment (**a**), which results in new proof states (child nodes). The network’s value is used to intelligently guide the search, focusing computational effort on the most promising paths (for example, by exploring the left branch more deeply).

We model the interactive proving process within Lean as a sequential decision-making problem, a standard formulation for RL tasks, in a similar way to refs. ^13,14^. To distinguish our formal RL task from the Lean proof assistant itself, we term this specific formulation the ‘Lean environment’. Each mathematical statement to be proved constitutes a distinct problem instance. We now formally define this environment using the standard RL terminology of states, actions, rewards and returns. At any time step *t*, the state *s*_*t*_ is the logical state of the Lean prover, encompassing established hypotheses and remaining goals, observed by the agent as the Lean tactic state (Fig. 1a, left). The agent interacts by proposing an action *a*_*t*_, a Lean tactic, as a text string. The environment attempts to execute these tactics, transitioning to a new state by updating hypotheses and goals (Fig. 1a, right). Each episode starts with a new problem statement and ends when a proof of that statement is successfully found, or a timeout occurs. The agent is incentivized to find short proofs by a reward signal *r*_*t*_ = −1 for each tactic applied. The return *G*_*t*_ from a state *s*_*t*_ is the sum of these rewards until termination. Crucially, for proof states that decompose into multiple independent subgoals that must all be solved, the return is defined as the minimum return over these subgoals (that is, corresponding to the longest proof branch), rather than the more natural sum of returns from each subgoal (see Methods for full RL formulation and value function definition).

### Prover agent

The AlphaProof agent combines a deep neural network with a powerful search algorithm inspired by AlphaZero^2^. At its core is the proof network, a 3-billion-parameter encoder–decoder transformer model^15,16^, that learns to interpret the observed Lean tactic state (Fig. 1b) and generate two outputs: a policy, suggesting promising tactics to apply next, and a value function, estimating the expected return *G*_*t*_ (as defined in the previous section). These outputs guide a specialized tree search that executes sequences of tactics and evaluates their consequences (Fig. 1c). Key adaptations for formal theorem proving include an AND–OR tree structure to handle the decomposition of proofs into multiple independent subgoals, similar to ref. ^17^, that must all be solved. Furthermore, to manage the large, open-ended space of possible tactics, AlphaProof samples actions^18^ and incorporates progressive sampling^19^ to explore a broader range of proof strategies along critical paths (see Methods for full details on the network architecture and tree search algorithm).

### Training

AlphaProof’s capabilities are primarily developed through a multi-stage training process. First, the proof network undergoes pretraining on a large corpus of approximately 300 billion tokens of code and mathematical text using a next-token prediction objective, a standard technique for language models. The goal of this stage is to imbue the network with a broad understanding of logical structures, programming syntax and mathematical language—an essential foundation for the subsequent stage to effectively learn from much smaller formal datasets. Next, supervised fine-tuning is performed using approximately 300,000 state–tactic pairs extracted from human-written proofs in the Mathlib library^4^. This stage enables the proof network to understand Lean syntax and internal states, imitate expert Lean tactics, and provide initial estimates for proof difficulty.

**Fig. 2:**
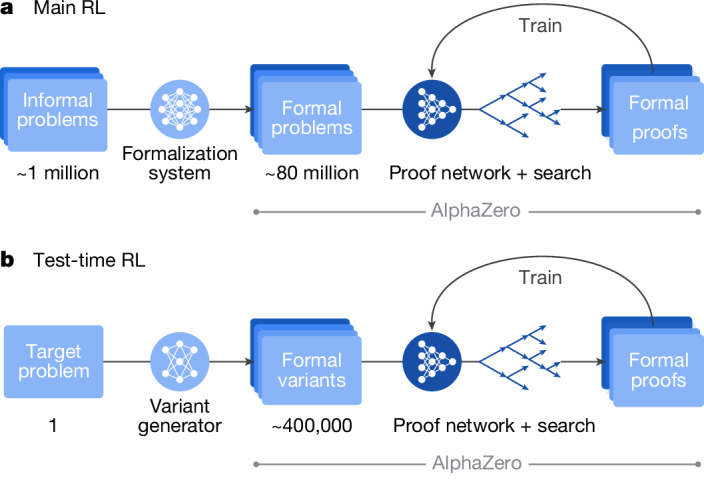
AlphaProof learning and adaptation processes. **a**, The main RL loop. Approximately 1 million informal mathematical problems are first translated into a large-scale (approximately  80 million) formal problem dataset by a formalization system. This dataset serves as the curriculum for an AlphaZero-like RL process. Here the proof network, in conjunction with the tree search, interacts with the Lean environment to generate formal proofs and disproofs. The experience gained from these attempts is used to iteratively train and improve the proof network, enhancing its ability to solve a broad range of mathematical problems. **b**, The TTRL loop. For a specific, challenging formal problem, the variant generator generates a diverse set of relevant formal variants. A focused AlphaZero-like RL process, again utilizing the proof network and tree search, then trains on this bespoke curriculum of variants. This targeted learning adapts the proof network to the specific structures and difficulties of the original hard problem, often enabling it to find a formal proof that was previously intractable. This process can be run on multiple target problems at the same time. Figure adapted from ref. ^28^, Google DeepMind.

The central learning phase, inspired by AlphaZero, is the main RL loop in which AlphaProof learns from self-generated experience. Unlike board games, however, the proving task lacks a single, universal initial state from which all other states can be derived through self-play. Consequently, learning to reason across diverse mathematical domains necessitates a vast and varied corpus of problem statements to serve as distinct starting points for the RL agent. While human-generated mathematical problems provide a natural source for such a corpus, the number of problems manually formalized in Lean is many orders of magnitude smaller than those available in natural language. To bridge this gap, we developed an auto-formalization process (Fig. 2a, left, and Extended Data Fig. 2). This process uses a Gemini-based LLM, fine-tuned and iteratively refined using human and synthetic data. Over the course of the project, this model auto-formalized approximately 1 million natural-language problems into a dataset of around 80 million formal Lean problems, vastly exceeding the scale of all other available datasets. Importantly, each auto-formalized statement, regardless of its fidelity to the original natural-language problem, provides a valid formal problem that AlphaProof can attempt to prove or disprove, thus serving as a useful training instance.

A matchmaker system assigns auto-formalized problems and adaptive compute budgets to distributed actors, randomly tasking them to either prove or disprove each statement. Actors then generate attempts in the Lean environment using a tree search algorithm that operates over proof trees and is guided by the current proof network. Lean-verified outcomes—whether a proof, a disproof or a timeout—provide grounded feedback. The proof network is continually improved (Fig. 2a, right) using experience from both successful proof and disproof attempts, refining its policy to predict effective tactics and adjusting its value to estimate the expected return. This continuous improvement based on self-generated experience allows AlphaProof to move far beyond imitating existing proofs, and discover potentially complex reasoning strategies necessary to solve challenging problems (see Methods for full details on each training stage, auto-formalization, the matchmaker and a summary of the required computational budget).

### Inference

When presented with a new problem, AlphaProof leverages two complementary computational scaling mechanisms, both initialized from its main RL-trained agent. First, increasing the tree search budget allows for a more exhaustive exploration of proof paths, a technique proven to be effective in the AlphaZero lineage^2^. Second, for problems where extensive search may be insufficient, AlphaProof uses a novel test-time RL (TTRL) approach (Fig. 2b). TTRL uses the same core AlphaZero-inspired RL algorithm as the main training phase (Fig. 2a), but instead of learning from a broad curriculum of auto-formalized problems, TTRL focuses learning on a bespoke curriculum of synthetic problem variants (for example, simplifications or generalizations) generated specifically around the target problem (Fig. 2b). TTRL thus allows the agent to dedicate substantial resources to learn problem-specific strategies, often unlocking solutions intractable through scaling up the tree search alone (see Methods for full details).

## Benchmarks

We evaluated AlphaProof on a comprehensive suite of formal mathematics benchmarks, all manually formalized in Lean, spanning advanced high-school to elite Olympiad and university-level problems. Our evaluation suite comprises: (1) a corrected version of the publicly available miniF2F benchmark^20^ (high-school mathematics competitions); (2) formal-imo, a benchmark of all non-geometry (because of specific Mathlib library limitations for Olympiad-style geometry; see ‘IMO-style geometry’ in Methods) historical IMO problems internally formalized by experts; and (3) the public Putnam benchmark^21^ (undergraduate Putnam Mathematical Competition problems). For the Putnam benchmark, problems from even-numbered years, 1990 onwards, were reserved as a dedicated held-out test set (PutnamBench-test). Rigorous data separation was maintained throughout all training phases to ensure evaluation of generalized reasoning capabilities rather than memorization (see Methods for details on data curation and separation). Together, these benchmarks form a standardized, diverse and demanding testbed for assessing AlphaProof’s capacity for advanced mathematical reasoning and problem solving.

## Main RL progress

**Fig. 3:**
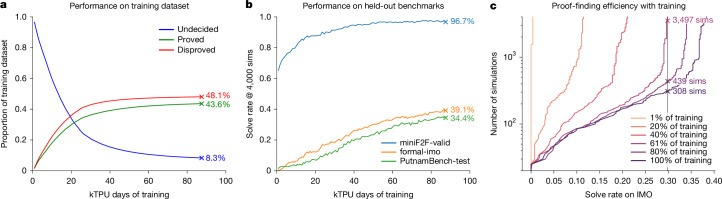
AlphaProof’s learning progression during main RL. **a**, Evolution of performance on the training dataset (auto-formalized problems). The lines show the proportion of starting problem instances that are proved (green), disproved (red) or remain undecided (blue) during main RL, as a function of compute (on Google Cloud ‘TPU v6e’s). **b**, Solve rate (at 4,000 simulations (sims)) on held-out benchmarks (miniF2F-valid, formal-imo and PutnamBench-test) throughout training. **c**, Number of tree search simulations (logscale) required to achieve a given solve rate on the historical IMO benchmark at different stages of main RL training. Each curve represents a checkpoint of the AlphaProof agent at a different stage of its main RL training. The plot shows that later-stage agents are not only stronger but also more efficient, requiring substantially less search to achieve the same solve rate as earlier agents. For instance, the final agent solves approximately 30% of problems with only 300 simulations, a level of performance that earlier agents could not reach even with vastly more search.

We analyse the performance of AlphaProof over the course of its main RL phase, which spanned approximately 80,000 tensor processing unit (TPU) days of training (equivalent to, for instance, 4,000 TPUs utilized for 20 days). In this stage, AlphaProof learns from self-generated experience by attempting to prove or disprove millions of problems drawn from its auto-formalized dataset. Throughout this training, the proportion of problems successfully proved or disproved steadily increases, leaving only a fraction of its dataset undecided, demonstrating mastery over its training curriculum (Fig. 3a). This learned capability generalized robustly to unseen problems; AlphaProof’s solve rate on held-out benchmarks (miniF2F-valid, formal-imo and PutnamBench-test) consistently improved, starting from the performance of the initial supervised fine-tuned model (the 0-TPU-day point in Fig. 3b) to a final performance that was far beyond other provers. Furthermore, the main RL phase significantly enhanced proof-finding efficiency. As training progressed, AlphaProof required substantially fewer tree search simulations to achieve a given solve rate (Fig. 3c), indicating that the neural network became a much stronger guide, internalizing effective proof strategies. This demonstrates how the large computational cost of RL training is transformed into inference-time efficiency, producing a more powerful and more efficient agent.

## Inference-time scaling

**Fig. 4:**
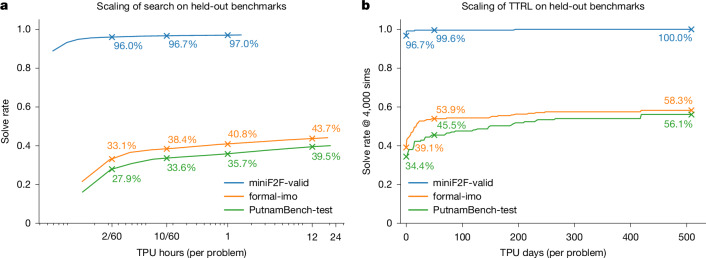
AlphaProof performance scaling with inference compute per problem. Solve rates on held-out benchmarks (miniF2F-valid, formal-imo and PutnamBench-test). In both panels, the ‘compute per problem’ is an average, calculated as the total TPU compute consumed during the evaluation, divided by the total number of problems in the benchmark. **a**, Solve rates as a function of increasing tree search compute per problem, measured in v6e TPU hours (logarithmic scale). Solve rates are highlighted for low search budgets (for example, 2/60 TPU hours per problem, corresponding to 2 minutes on 1 TPU) and more extensive search. **b**, Scaling with TTRL compute. Solve rates as a function of increasing TTRL training compute per target problem, measured in v6e TPU days (linear scale). Solve rates are highlighted after an initial TTRL compute investment (for example, 50 TPU days or 1 day on 50 TPUs per problem) and at the end of the TTRL phase with performance evaluated using 4,000 simulations. Note the different *x*-axis units and scales (logarithmic TPU hours versus linear TPU days) between panels.

Once main RL training is complete, AlphaProof’s performance on unseen problems can be further enhanced by allocating additional computational resources at inference. We explore two complementary scaling mechanisms operating on different timescales (Fig. 4). First, increasing the tree search budget yielded significant improvements in solve rates across all benchmarks (Fig. 4a). The agent’s learned search policy enabled a strong performance baseline even with low inference budgets (for example, 2 minutes per problem on 1 TPU). Extending this search budget progressively to several TPU hours allowed for consistently increased solve rates. For instance, scaling the search from 2 TPU minutes to 12 TPU hours boosted solve rates by over 10 absolute percentage points on the formal-imo and PutnamBench-test benchmarks. We observe similar scaling properties on PutnamBench-train (Extended Data Table 2). For problems remaining unsolved even with extensive tree search, AlphaProof uses TTRL to enable deeper, problem-specific adaptation (Fig. 4b; see Methods for the TTRL procedure). This approach yielded rapid initial gains, solving many new problems within the first 50 TPU days, and continued to find new solutions as training progressed over longer durations. This sustained learning substantially elevated performance beyond tree search scaling alone, increasing solve rates by an additional 15 absolute percentage points on both formal-imo and PutnamBench-test compared with a 12 TPU hours search (Fig. 4b and Table 1). The efficacy of TTRL itself is also influenced by factors such as variant quality and quantity (Extended Data Fig. 3). The distinct *x*-axis scales in Fig. 4 (TPU hours for search versus hundreds of TPU days for TTRL) highlight the different computational investments and capabilities of these two complementary scaling strategies, with TTRL providing a powerful mechanism for tackling the most challenging problems. A detailed breakdown of both tree search and TTRL scaling by mathematical subject for the formal-imo and PutnamBench-test benchmarks, illustrating the dynamic improvement with increasing compute, is provided in Extended Data Fig. 4.

**Table 1:** Performance of AlphaProof on formal mathematics benchmarks

Name	Compute budget (per problem)	miniF2F-test^20^	formal-imo	PutnamBench-test^21^
**Previous state of the art (before IMO 2024)**				
GPT-F expert iteration^24^	–	36.6%	–	–
Hypertree proof search^17^	–	41.0%	–	–
InternLM2-Math-Plus-7B^23^	–	43.4%	–	–
**Previous state of the art (after IMO 2024)**				
Kimina-Prover Preview^25^	–	80.7%	–	1.6%
DeepSeek-Prover-V2^26^	–	88.9%	–	5.3%
**This Work**				
AlphaProof	2 TPU minutes	96.3%	33.2%	27.9%
AlphaProof	12 TPU hours	97.7%	43.7%	39.4%
AlphaProof with TTRL	50 TPU days	97.5%	53.9%	45.5%
AlphaProof with TTRL	500 TPU days	99.6%	58.3%	56.1%

AlphaProof is compared against other methods, using the strongest reported result for each system, corresponding to their largest compute budgets. Cells with ‘–’ indicate unavailable compute budgets or evaluation results. For AlphaProof, the reported ‘compute budget (per problem)’ refers to the average computational cost as defined in Fig. 4a and is an inference-time budget only that does not include the amortized cost of the main RL training loop. AlphaProof’s miniF2F-test results are reported on a corrected version of the dataset (see Methods for details). Results for other methods on miniF2F-test are as reported in their respective publications, based on the dataset versions they utilized; direct comparison should therefore be made with caution, considering potential dataset differences. For PutnamBench-test, scores for previous work were recalculated based on the publicly available proofs reported by each system, for consistent comparison against our PutnamBench-test split; these may differ from figures reported by those systems for the full PutnamBench.

## Final benchmark evaluation

The comprehensive training and inference scaling strategies culminate in AlphaProof establishing a state-of-the-art performance across all evaluated formal mathematics benchmarks (Table 1).

AlphaProof shows strong performance even at modest compute budgets, achieving state-of-the-art results on several benchmarks even with just 2 TPU minutes of search budget per problem (Fig. 4a and Table 1). This efficiency is particularly notable on PutnamBench-test, where it substantially outperforms previous systems, underscoring its strong foundational reasoning capabilities and its effectiveness in resource-constrained scenarios. For peak performance on the most complex problems, TTRL is crucial, extending the state of the art by a significant margin (Fig. 4b). This approach yields comprehensive gains across all benchmarks (Table 1), with a perfect score on miniF2F-valid (Extended Data Table 2) and near-perfect scores on the test split. On formal-imo, TTRL reached particularly strong performance in number theory (75.7%) and algebra (72.6%), and also made progress in combinatorics (20.3%). Similar comprehensive gains were observed on the PutnamBench-test. Detailed subject performance is presented in Extended Data Fig. 3 and Supplementary Table 1. A few proofs are also shown in Supplementary Information.

## Performance at the 2024 IMO

The IMO is the world’s most prestigious pre-collegiate mathematical competition. Held annually since 1959, each participating country sends a team of up to six elite students, selected through rigorous national competitions. Contestants face six exceptionally challenging problems across algebra, combinatorics, geometry and number theory, designed to test deep conceptual understanding and creative problem solving, pushing them far beyond standard curricula and often requiring hours of intense thought. Achieving an IMO medal is a significant honour, often marking the early careers of individuals who later become leading mathematicians.

To assess AlphaProof’s capabilities on an unseen competition, we applied it to the problems from the 2024 IMO, operating as the core reasoning engine within a complete problem-solving pipeline. This version of AlphaProof was developed until July 2024 using a similar training and inference methodology to that described above. Given specific Mathlib library limitations for Olympiad-style geometry (‘IMO-style geometry’ in Methods), the geometry problem (P4) was addressed using the specialized AlphaGeometry 2 system^3^. The remaining five non-geometry problems (algebra, P1 and P6; number theory, P2; combinatorics, P3 and P5) were manually formalized in Lean by experts immediately after the competition’s release (‘IMO 2024 evaluation protocol and methods’ in Methods). Several problems (P1, P2, P5 and P6) required identifying the answer (for example,‘Find all functions…’) before constructing a proof. For these, hundreds of candidate answers were generated by querying the public Gemini 1.5 Pro model with Python tool use enabled and a collection of hand-written examples given as a few-shot prompt^22^, successfully guessing the correct answers for all applicable problems. AlphaProof then demonstrated high efficiency by rapidly refuting the vast majority of these incorrect candidates, isolating the correct ones for full proof attempts. Subsequently, using TTRL, AlphaProof successfully discovered formal proofs for all algebra and number theory problems: P1, P2 and P6 (proofs in Extended Data Figs. 7–9). Each of these solutions required 2–3 days of TTRL, demonstrating substantial problem-specific adaptation at inference. The two combinatorics problems (P3 and P5) remained unsolved by our systems.

**Fig. 5:**
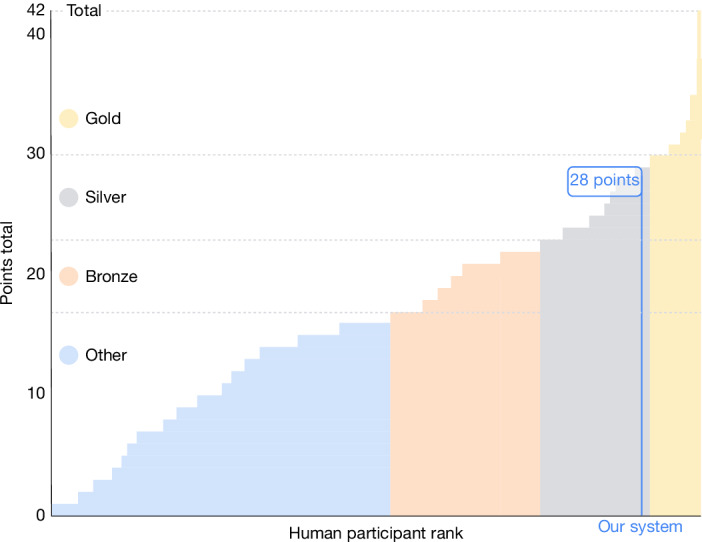
Combined AI system performance at the IMO 2024. Our combined system scored 28 points, with AlphaProof solving three problems (P1, P2 and P6) and AlphaGeometry 2^3^ solving one (P4). This places the performance of our system relative to human competitors^27^ within the silver-medal threshold. Official medal boundaries (gold, silver, bronze) are shown. Figure reproduced from ref. ^28^, Google DeepMind.

The combined system, with AlphaProof solving P1, P2 and P6, and AlphaGeometry 2 solving P4, successfully solved four of the six IMO problems. This yielded a total score of 28 out of 42 points (‘Discussion and conclusion’), placing our AI system’s performance within the silver-medal range of the official competition, one point below the gold-medal threshold (Fig. 5). Although the multi-day computational effort for AlphaProof’s solutions significantly exceeds the time constraints faced by human contestants, it is crucial to note that before this work, even the most advanced AI systems could typically solve only a small fraction of the easiest historical IMO problems^13,17,23^. AlphaProof’s success in solving three problems from a live IMO competition, including 2024’s most difficult P6, which was solved by only five human contestants, therefore demonstrates a profound advance in the potential of AI for mathematical reasoning, even when applied to problems renowned for their difficulty and requirement for novel insights.

## Discussion and conclusion

We have introduced AlphaProof, an RL agent capable of proving complex mathematical problems within the formal environment of the Lean theorem prover. By combining an AlphaZero-inspired learning framework with a curriculum of millions of auto-formalized problems and a TTRL approach based on variant generation, AlphaProof has demonstrated a powerful capacity for automated mathematical reasoning. Our results show that AlphaProof significantly advances the state of the art across a diverse suite of formal mathematics benchmarks, including those derived from historical IMO and Putnam competitions. The capabilities of this system were notably demonstrated at the 2024 IMO. As part of a complete problem-solving pipeline, AlphaProof successfully solved the three algebra and number theory problems, including the most difficult problem P6, and AlphaGeometry 2 solved the geometry problem. This combined performance resulted in solving four of the six competition problems, thereby reaching the same score as a 2024 IMO silver medallist. To our knowledge, this marks a landmark achievement as the first time an AI system has attained any medal-level performance at the IMO, providing compelling evidence of the sophisticated reasoning abilities emerging from learning from grounded, verifiable experience at scale.

Although AlphaProof’s results represent a significant step, several limitations and avenues for future development remain. Although open-source pre-trained foundation models are now available, the bespoke learning phase required by AlphaProof represents a scale of domain-specific training that is likely beyond the reach of most academic research groups. Furthermore, the multi-day inference time required by TTRL for solving the most difficult problems, although effective, highlights the need for more efficient inference-time strategies. Future progress will probably involve continued algorithmic and engineering refinements, further advancements in auto-formalization, and more optimized strategies for TTRL.

Furthermore, AlphaProof’s current successes are primarily within the domain of advanced high-school and undergraduate competition mathematics. Although exceptionally challenging, these problems operate within a known fixed library of mathematical concepts with a certain degree of thematic consistency. Our TTRL approach proved to be highly effective in this setting, and understanding its generalization is part of the considerable next step of extending these capabilities to the frontiers of research mathematics. This transition is particularly challenging as it requires moving beyond pure problem solving to theory building—the continuous expansion of this library with new concepts. Ultimately, imbuing AI with an understanding of mathematical taste, interestingness or beauty, remains a fascinating open research question.

The significant computational requirements of this work raise important questions about accessibility and the future of an open research ecosystem. We believe this work is best viewed as foundational, pathfinding research; demonstrating that a certain capability is achievable often requires a scale of investment that can later be optimized and democratized. To that end, and to ensure our work serves as a community catalyst rather than a barrier, we are taking concrete steps such as providing an interactive tool for exploration. A key direction for our future research is to substantially improve algorithmic efficiency, with the explicit goal of lowering the computational barriers to entry and ensuring that these techniques can become a collaborative tool for the entire mathematical community.

Despite the challenges mentioned above, we anticipate that continued development, particularly in addressing computational and scope limitations will pave the way for AlphaProof to become an increasingly valuable collaborator for human researchers in exploring the frontiers of mathematics and, by extension, other scientific disciplines.

## Methods

### Related work

#### Interactive theorem proving

Our research is grounded in the field of interactive theorem proving, where humans formalize theorems and construct verifiable proofs using proof assistants such as Isabelle^29^, HOL Light^30^, Rocq^31^ and Lean^1^. These tools have enabled significant achievements, including the formal verification of the four-colour theorem^32^ and critical software such as the CompCert C compiler^33^. However, such successes typically demand extensive work from domain experts. AlphaProof aims to mitigate this bottleneck by automating aspects of both theorem formalization and proving, thereby reducing the requisite labour and expertise.

#### Machine learning for theorem proving

The intersection of machine learning and theorem proving has seen significant advancements, particularly with the advent of LLMs^34–36^ inter alia, and the availability of formalized mathematics benchmarks such as MiniF2F^20^, ProofNet^37^, PutnamBench^21^, LISA^38^ or HOList^39^. Our work builds upon these developments.

An automated feedback signal from theorem provers for correct proofs has been pivotal for applying large-scale machine learning to theorem proving. Early research^13,24,40,41^ explored generative language modelling for automated theorem proving, learning from human-generated proofs and incorporating techniques such as expert iteration or RL. Concurrently, several studies^14,42–44^ have focused on jointly training tactic prediction with premise selection. In contrast to these step-by-step and search-based methods, Baldur^45^ pioneered whole-proof generation, using an LLM to produce a complete proof at once. Notably, ref. ^46^ introduced an approach that guided formal proving with informal proofs (‘draft, sketch and prove’ methodology). This line of research is further extended by work on subgoal decomposition^47^. More sophisticated search strategies have also been explored in neural theorem proving, for example, HyperTree Proof Search^17^ and variants of Monte Carlo tree search^48,49^. Very recently, Kimina^25^ and DeepSeek-Prover^26^ explored generating the interleave of natural-language explanations and formal proofs via LLMs.

#### Auto-formalization of mathematics

A critical component of bridging the gap between human-written mathematics and formal theorem provers is auto-formalization. This area focuses on automatically translating natural-language mathematical statements into formal proofs or formalized statements. Significant progress includes early work^50,51^, and, more recently, LLM-based approaches for this challenging task^52–54^. Subsequent research used synthetically generated data to fine-tune LLMs and enhance their auto-formalization capabilities^49,55,56^. More recently, contributions have addressed the evaluation of auto-formalizations^57,58^. Our work extends these research directions by developing an auto-formalization specialist that generates millions of formal statements for our prover agent.

#### Reasoning with LLMs

Beyond dedicated theorem provers, the broader field of reasoning with LLMs has seen substantial progress. Techniques such as chain-of-thought prompting^59^ and RL, often coupled with planning, have significantly boosted LLM reasoning capabilities, especially in mathematical and coding tasks^60^. More recently, OpenAI O1^61^ and DeepSeek R1^9^ have demonstrated the effectiveness of scaling RL for LLM reasoning, observing logarithmic scaling with compute at both training and test time. This approach has led to gold-equivalent performance at the International Olympiad in Informatics^62^ and strong performance in the American Invitational Mathematics Examination. However, the IMO has so far remained out of reach for current LLMs, with recent studies highlighting their limited proving capabilities^11,12^.

#### TTRL

Our methodology extends on trends in test-time compute scaling^9,61–63^ and adaptation^64–66^, including early TTRL in games^67,68^. Distinct from these approaches, and concurrent TTRL methods using majority-vote rewards for unlabelled informal mathematics^69^ or numerical verification for recursively simplified integration variants^70^, AlphaProof’s TTRL methodology (first outlined in ref. ^28^) uniquely addresses elite-level formal mathematical problem solving, particularly in the domain of competition mathematics. It achieves this by generating a diverse curriculum of relevant formal variants for challenging IMO and Putnam-level problems within Lean, and leveraging Lean’s rigorous verification to guide learning, ultimately enabling the discovery of complex, validated proofs in this formal setting.

### AlphaProof

AlphaProof is an RL agent designed to discover formal mathematical proofs by interacting with a verifiable environment based on the Lean theorem prover^1^. The system integrates several core technical components necessary for tackling this complex domain: an RL environment defining the theorem-proving task (the Lean environment), a deep neural network architecture capable of representing proof states and suggesting proof continuations (proof network), a specialized tree search algorithm adapted for formal proofs, a translation process for generating a large-scale problem dataset for training (auto-formalization), a large-scale RL phase to discover general proof strategies (main RL), and a focused learning procedure for difficult instances (TTRL). The specifics of each component are described below.

### Lean environment

**Extended Data Fig. 1:**
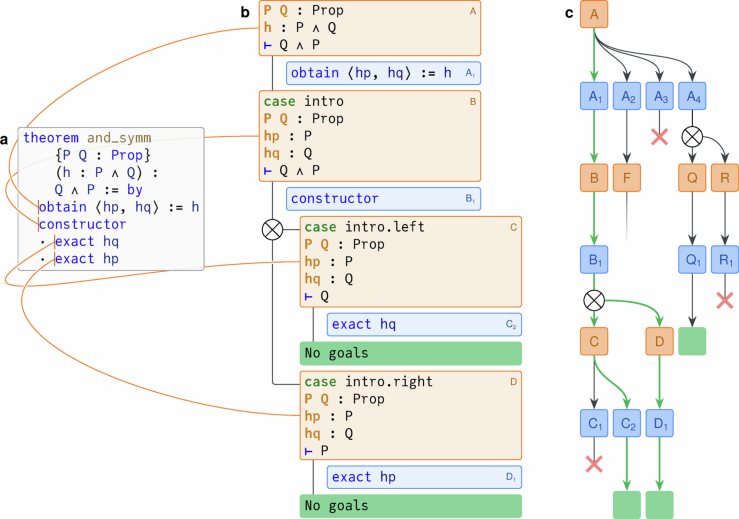
The Lean tactic environment and its framing for tree search. **a**, The Lean editor environment, with tactic state information associated with cursor positions. **b**, A corresponding proof tree, linking observations (tactic states) through actions (tactics). The ⊗ symbol indicates where there are multiple subgoals which must all be solved. **c**, The search tree, with the successful proof found highlighted in thick green arrows.

*Formal mathematics as the RL environment*. Formal mathematics provides a rigorous framework for expressing mathematical statements and their proofs, enabling automated verification of correctness. We utilize the Lean interactive theorem prover^1^, which is built upon a dependent-type theory known as the calculus of inductive constructions^71^. In this paradigm, mathematical statements are represented as types, and a proof corresponds to constructing a term that is of (inhabits) that type. For instance, the theorem ∀ P Q, P ∧ Q → Q ∧ P is proven by constructing a function term, such as fun P Q ⟨hp, hq⟩ ↦ ⟨hq, hp⟩, that inhabits the corresponding type. Although it is ultimately this ‘term mode’ that Lean’s trusted kernel consumes to verify the correctness of a proof, Lean also offers a higher-level ‘tactic mode’, which saves the user from constructing these terms by hand. Tactics are tools that construct the underlying proof term by manipulating the proof state, which consists of current hypotheses and goals. For example, the aforementioned theorem can also be proven using a sequence of tactics such as intro P Q h; simp [h]. During tactic-mode proving, Lean provides contextual information via a ‘tactic state’ display (Fig. 1a and Extended Data Fig. 1a), showing current goals and their associated hypotheses. It is this tactic mode of Lean that we cast as an RL environment (Extended Data Fig. 1b).

States (*s*_*t*_): a state represents the complete logical context within the Lean prover at a given step *t* of a proof attempt, encompassing all active hypotheses and remaining goals.

Observations: the agent observes the state as a pretty-printed string representation of the Lean tactic state.

Actions (*a*_*t*_): an action is a Lean tactic, represented as a text string.

Transitions: applying an action *a*_*t*_ to state *s*_*t*_ involves the Lean environment attempting to execute the tactic. If successful and valid (see ‘Tactic validation and execution’), this results in a new state *s*_*t*+1_ with updated hypotheses and/or goals.

Episodes: each episode commences with a new mathematical statement (the initial state) to be proven. An episode terminates when: (1) a complete, Lean-verified proof of the initial statement is found; or (2) a predefined computational budget is exhausted.

Reward signal and objective: to incentivize the discovery of the shortest proof, a reward of *r*_*t*_ = −1 accompanies each tactic applied.

Return definition: the return *G*_*t*_ from a state *s*_*t*_ is the sum of rewards until termination. For states that decompose into multiple independent subgoals (AND nodes, see ‘Goal decomposition’ in ‘Interfacing with Lean’), the return (and thus the value function target) is defined as the minimum return (that is, corresponding to the longest and hardest proof branch) among its constituent subgoals. This definition recursively applies to ancestor states of such multi-goal states. Although summing the returns from each subgoal, thereby minimizing the total number of tactics in the proof, is a natural alternative, our choice of the minimum return provides an interesting incentive as it encourages the agent to split goals into subgoals of balanced difficulty. This results in the value of a state to correspond to −*T*_steps_, where *T*_steps_ is the number of tactics in the longest branch of the proof required to resolve all subgoals.

*Interfacing with Lean*. To enable RL, AlphaProof interacts with a custom environment built upon the Lean 4 interactive theorem prover^1^. It enables executing tactics, handles proof states, implements required additional operations and incorporates optimizations for large-scale tree search.

*Core Lean and Mathlib integration*. The environment utilizes Lean 4 as its foundational proof assistant. The extensive Mathlib library^4^ is loaded, providing a wide array of mathematical definitions, established theorems and high-level tactics (for example, the linear arithmetic solver linarith) that the agent can leverage. To support the formalization of specific problem domains encountered (for example, IMO-style problems) and to provide general utility tactics not yet present in the main library at the time of development, a focused set of custom additions was developed. This included a small set of custom definitions, approximately 100 elementary theorems and specialized, reusable tactics for common symbolic manipulations (for example, for numeral and list simplification such as (3/4).num to 3). These additions were designed to be general purpose, addressing common patterns or missing low-level infrastructure, and critically, many have since been accepted and integrated into the public Mathlib library, underscoring their broad utility and alignment with the library’s development goals.

*Environment operations*. To support the demands of AlphaProof, our environment enables the following.

Parallel execution and state management. The environment is designed to manage and operate on multiple Lean proof states concurrently across multiple threads. This allows for parallel simulations within the tree search and efficient batching of queries to the proof network. Mechanisms are implemented to save, restore and uniquely identify Lean tactic states, enabling the tree search to resume search from any previously visited state.

Tactic validation and execution. When the proof network proposes a tactic (as a text string), the environment attempts to execute it within the current Lean proof state. For a tactic application to be considered successful, it must not only execute without error but also the resulting proof term must not employ the ‘sorry’ placeholder used for incomplete proofs, and moreover must be type-correct. To evaluate the latter, goals not directly addressed by the tactic are temporarily ‘closed’ using a private ‘internalSorry’ axiom, in a way that also captures intermediate claims generated by tactics such as ‘have’. The ‘private’ keyword limits the axiom’s visibility to discourage its use outside this specific tactic. Ultimate soundness is guaranteed by the resulting closed term being verified by the Lean kernel (see ‘Interfacing with Lean’).

Goal decomposition (AND-node state splitting). If a tactic application successfully decomposes the current goal into *N* > 1 independent subgoals (for example, proving *P* ∧ *Q* splits into proving *P* and proving *Q*), the environment splits the current Lean tactic state into *N* distinct child states. In each of these child states, all but one goal are assigned with ‘internalSorry’ to leave a single open goal. This allows each subgoal to be treated as an independent problem by the tree search, forming the basis for AND-node handling in the search algorithm (see ‘Tree search algorithm’).

Disproving mechanism. To enable the agent to disprove statements (that is, prove their negation), the environment provides an operation to transform a goal into its negation. This is achieved by applying a custom tactic that utilizes a private axiom internal_true_if_false : (*α* → False) → *α*. This tactic reverts all local hypotheses, applies the axiom and performs clean-up of negated quantifiers, thereby establishing a new context for disproving the original statement, containing exactly the negation of the fully quantified goal we were previously in, and do so in a way that still lets our final proof be type-checked by the kernel (see ‘Interfacing with Lean’).

*Performance and stability considerations*. Several modifications were implemented to ensure that the Lean environment could operate efficiently and robustly under the high throughput demands of RL training. In particular:

- Key Mathlib tactics frequently used by the agent (for example, linarith) were compiled to C code. This results in some tactics executing (that is, generating proof terms) six-times faster.
- A wall-clock time limit was imposed on individual tactic executions, complementing Lean’s internal ‘heartbeat’ limit for computational steps.
- Lean’s internal checkSystem function, which monitors resource consumption, was invoked more frequently within critical code paths to allow for earlier, safe abortion of overly long or resource-intensive tactic applications.
- The multi-precision integer library within Lean was modified to enforce a hard cap on the size of numerals, preventing runaway computations with extremely large numbers.

*Proof verification final check*. To provide absolute assurance of correctness (assuming the soundness of the Lean kernel and the declared axioms), every proof or disproof found by AlphaProof undergoes a final, independent verification step. This involves executing the standard Lean command-line tool on a .lean file containing the complete theorem statement and the agent-generated proof. A custom command also verifies that the proof only relies on the three commonly accepted axioms built-in to Lean itself (propositional extensionality, global choice and soundness of quotient types).

### Prover agent

*Proof network*. The proof network is a 3-billion-parameter encoder–decoder transformer model^15^, similar to ref. ^16^. The encoder takes the pretty-printed Lean tactic state as input and outputs a latent representation of the current proof state. This latent representation serves as input to two distinct heads: the decoder, which acts as the policy and generates *K* tactics in parallel at inference, and a value head situated atop the encoder. Consistent with previous work^72^, the value head parameterizes the value as a categorical distribution, estimating the expected return. Key architectural hyperparameters are summarized in Supplementary Table 2.

*Tree search algorithm*. The proof network guides a tree search algorithm to explore the space of possible proof constructions. The tree search is adapted from AlphaZero^2^ and Sampled MuZero^18^, incorporating several key modifications for formal theorem proving. The search tree consists of nodes representing Lean tactic states and edges representing tactics applicable at those states (Extended Data Fig. 1c). Each search iteration involves three phases: selection, expansion and backpropagation. Key hyperparameters for the search are provided in Supplementary Table 3.

*Standard tree search framework*.

Selection. Starting from the root state, a path is traversed to a leaf node by recursively selecting an action *a* at each state *s* that maximizes a probabilistic upper confidence tree (PUCT) bound^73,74^, adapted from ref. ^2^:

$$Q(s,a)+c(s){\pi }^{1/\tau }\,(a| s)\frac{\sqrt{{\sum }_{b}N(s,b)}}{N(s,a)+1}.$$

Here, *N*(*s*, *a*) is the visit count for action *a* from state *s*. The prior *π*^1/*τ*^(*a*∣*s*) is derived from the proof network’s policy output, modified by a temperature *τ*. The exploration factor *c*(*s*) is given by

$$c(s)={c}_{{\rm{i}}{\rm{n}}{\rm{i}}{\rm{t}}}+\log \left(\frac{N(s)+{c}_{{\rm{b}}{\rm{a}}{\rm{s}}{\rm{e}}}+1}{{c}_{{\rm{b}}{\rm{a}}{\rm{s}}{\rm{e}}}}\right).$$

Whereas in previous work^72^, the values *Q*(*s*, *a*) were normalized to [0, 1] using the maximum and minimum estimated values of all children in state *s*, in AlphaProof we set

$$Q(s,a)={\gamma }^{-V(s,a)-1},$$

where *γ* is a discounting factor and *V*(*s*, *a*) is the aggregated search value, which has the semantic of the negative number of steps left to resolve the current goal. For state–action pairs that have not been visited in the search already, we set *V*(*s*, *a*) to be equal to the network prediction for the parent node *V*(*s*) minus a fixed penalty.

Expansion. When a simulation reaches a leaf node *s*_L_ not previously expanded, *K* tactics are sampled from the proof network’s policy *π*(⋅∣*s*_L_). Each tactic is validated in the Lean environment; invalid tactics are discarded; tactics that lead to the same Lean state (up to renaming and reordering of syntactically identical hypotheses) are merged together, keeping the one that minimizes a cost function linearly depending on string length and execution time. The newly expanded node’s value *V*(*s*_L_) is estimated by the proof network.

Backpropagation. The value estimate from the expanded leaf node is backed up along the selected path, updating the *N*(*s*, *a*) and *V*(*s*, *a*) statistics for all traversed state–action pairs.

*Key adaptations for AlphaProof*.

Progressive sampling. To dynamically broaden the search in promising regions, progressive sampling is used. If a node *s* encountered during a simulation path satisfies *n*(*s*) ≤ *CN*(*s*)^*α*^ (where *n*(*s*) is the number of times tactics have previously been sampled at *s* and *N*(*s*) its total visit count), an additional *K* tactics are sampled from *π*(⋅∣*s*) and added as new edges to *s*. This allows AlphaProof to explore a wider range of proof strategies along critical paths.

AND nodes for multi-goal states. To manage proof states with multiple independent subgoals (a logical AND condition), the tree search incorporates AND nodes, conceptually similar to ref. ^17^. An AND node is introduced when a tactic application decomposes a goal into several independent subgoals (Extended Data Fig. 1). Actions from an AND node correspond to selecting one of its child subgoal to focus on. During selection at an AND node, only unproven subgoals are considered for exploration. The PUCT formula is modified to prioritize the unproven subgoal perceived as most difficult (that is, having the largest *V*(*s*, *a*) or smallest *Q*(*s*, *a*)). This is achieved by replacing *Q*(*s*, *a*) in the standard PUCT formula with 1 − *Q*(*s*, *a*). The modified selection for an AND node *s*_AND_ acting on subgoal *a* is

$$(1-Q({s}_{{\rm{A}}{\rm{N}}{\rm{D}}},a))+{c}_{{\rm{A}}{\rm{N}}{\rm{D}}}c({s}_{{\rm{A}}{\rm{N}}{\rm{D}}})\pi (a| {s}_{{\rm{A}}{\rm{N}}{\rm{D}}})\frac{\sqrt{{\sum }_{b}N({s}_{{\rm{A}}{\rm{N}}{\rm{D}}},b)}}{N({s}_{{\rm{A}}{\rm{N}}{\rm{D}}},a)+1}.$$

The prior *π*(*a*∣*s*_AND_) is set to be uniform over all subgoals, and *c*_AND_ is an additional multiplier.

During backpropagation through an AND node, the value passed up is the minimum *V*(*s*_AND_, *a*) of its children subgoals, consistent with the definition of the return (see ‘Formal mathematics as the RL environment’).

Single search. For each proof attempt on an initial problem statement, AlphaProof executes a single search that terminates if a proof or disproof is found, or if the allocated simulation budget is exhausted. Unlike previous AlphaZero applications, AlphaProof does not commit to intermediate actions and does not restart the search from subsequent states within a single proof attempt. This is possible as theorem proving within a formal system is analogous to a single-player game in a perfectly simulated environment. The agent can retain and expand a single search tree over the entire proof attempt, allowing it to globally allocate its computational resources across all potential proof paths without needing to commit to any intermediate step.

### Training

*Pretraining and supervised fine-tuning*. Before RL, the AlphaProof proof network is initialized through a multi-stage training process.

First, the network is pre-trained on large datasets consisting of approximately 300 billion tokens of publicly available code and mathematical texts. This phase utilizes a next-token prediction objective with dropout and masked span reconstruction for regularization. The encoder processed approximately 12 trillion tokens whereas the decoder reconstructed approximately 3 trillion tokens, totalling roughly 50 epochs over the dataset. This stage imbued the policy head (decoder) with a broad understanding of programming syntax, logical structures and mathematical language.

Following pretraining, the model undergoes supervised fine-tuning (SFT). This stage utilizes a much smaller but more specialized corpus, containing approximately 300,000 state–tactic pairs amounting to approximately 5 million tokens of tactics, extracted from the human-authored proofs within the Mathlib library^4^. This process refines the policy head for Lean-specific tactic generation and initializes the value head to estimate the number of remaining steps in a proof, based on the structure of the Mathlib proofs. Hyperparameters for both pretraining and SFT are detailed in Supplementary Table 4.

**Extended Data Fig. 2:**
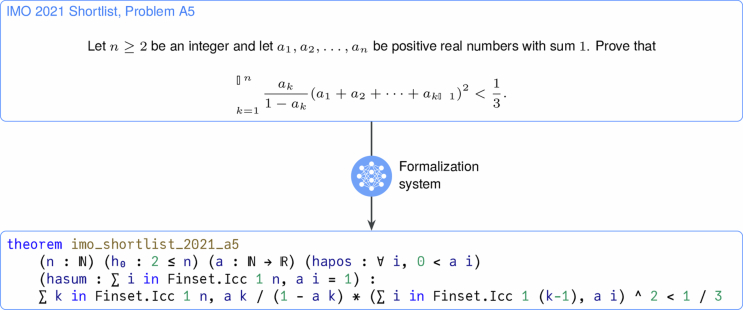
An example of the auto-formalization process. The formalization system in Fig. 2a receives the LaTeX-formatted natural language text of a problem statement, and outputs formal statements as valid Lean code. Credit: Google DeepMind.

**Extended Data Fig. 3:**
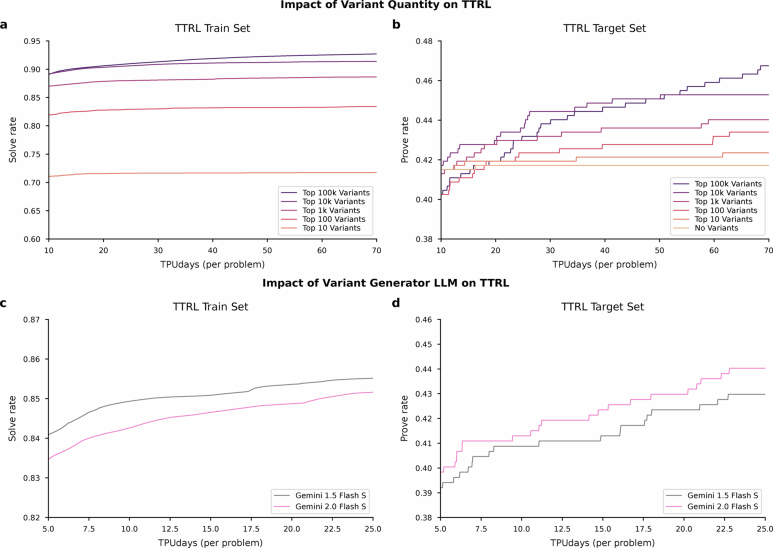
TTRL ablations when varying the quantity of synthetic variants and with different variant generator LLMs. **a** Increasing the number of variants in the curriculum (from “Top 10” to “Top 100k”) leads to a higher proportion of solved variants int the TTRL training set. **b** More synthetic variants consistently improves the final prove rate on the target problems, demonstrating the significant benefit of a larger set of problem-specific variants. **c** The variants in the TTRL train set from Gemini 1.5 Flash S (grey) show a higher solve rate than those from the stronger Gemini 2.0 Flash S (pink), suggesting Gemini 2.0 Flash S’s variants may form a more challenging learning curriculum. **d** Using Gemini 2.0 Flash S for variant generation results in a notably higher prove rate on the target problems, indicating that the learning curriculum is indeed more effective.

*Auto-formalization*. To provide a sufficiently large and diverse curriculum for RL, AlphaProof relies on an auto-formalization process. This process translates mathematical statements from natural language into the formal language of Lean (Extended Data Fig. 2), generating a dataset of approximately 80 million Lean problems from an initial set of roughly 1 million natural-language statements.

*Model and training data*. The auto-formalization system is built upon a specialized Gemini 1.5 Pro language model fine-tuned on a set of (natural-language problem statement, formalization chain-of-thought, Lean formal statement) triplets. Initial training data comprised approximately 2,500 problems, spanning pre-algebra to IMO-level difficulty, manually formalized in Lean by domain experts. For a subset of about 50 of these problems, experts also provided detailed chain-of-thought explanations outlining the formalization steps. This initial seed set was expanded to roughly 7,000 triplets by prompting the public Gemini 1.5 Pro model with the expert-annotated examples to generate additional chains-of-thought for the existing translations; low-quality synthetic chains-of-thoughts, identified by ad hoc formatting rule checks, were discarded.

*Iterative refinement*. To further enhance the formalization model’s accuracy, we used an iterative refinement strategy analogous to Self-Taught Reasoner (STaR)^60^. This process focused on a subset of problems for which ground-truth (‘golden’) Lean formalizations were available. The current fine-tuned model repeatedly generated chains-of-thought and Lean formalizations (see Supplementary Data 1 for the generation process). These outputs were automatically assessed for equivalence against the golden formalizations by tasking a low-compute version of AlphaProof to prove equivalence between the generated and golden statement. Concretely, the Lean statement AlphaProof was certifying was type_of% @generated = type_of% @golden. Traces leading to equivalence were added to the training data for subsequent fine-tuning iterations, ultimately expanding the high-quality triplet dataset to approximately 70,000 examples.

*Evaluation and benchmarking*. The performance of the auto-formalization model was regularly monitored through A/B studies. These studies compared a baseline model with a test model on two internal benchmarks: one comprising 50 representative IMO problems and another with 50 Putnam problems (Supplementary Table 5), both in natural language. For each problem, both models generated a single candidate formalization using a standardized procedure (Supplementary Data 1, incorporating majority voting, syntax checking, cycle consistency and rapid disproof/proof attempts by AlphaProof). These paired outputs were then evaluated by an internal panel of approximately three Lean experts for pointwise correctness and side-by-side preference.

Before the IMO 2024 competition, the final auto-formalization process achieved a 60% pass@1 success rate on the 50 representative IMO problems, with notable strengths in algebra (81.3%) and number theory (76.9%), and a lower rate for combinatorics (33.3%). On the 50 representative Putnam problems, the pass@1 rate was 64%, with strong performance in number theory (72.7%), algebra (61.9%) and geometry (54.6%). See details in Extended Data Table 1.

*Dataset generation*. Several key methods facilitated the generation of a training dataset suitable for formalizing a wide range of mathematical problems. To address problems requiring an answer (‘Find all *n*…’), plausible answers were gathered alongside questions and injected during auto-formalization. We also continuously augmented the dataset by reapplying the improved formalization pipeline to the source problems. Finally, this sampling process involved producing multiple distinct formal translations for each natural-language problem. This strategy ensured a higher likelihood of including a correct formalization, with incorrect ones often being quickly resolved during main RL or sometimes presenting interesting problems in their own right. This resulted in a large and diverse dataset of approximately 80 million formalized mathematical problems necessary for effective RL training.

*Main RL*. The main RL phase further trains the proof network, initialized from pretraining and SFT. The RL training architecture comprises three main components: a centralized matchmaker, distributed actors and a centralized learner. The RL training was conducted for approximately 1 million training steps (Supplementary Table 6).

*Start positions dataset*. The training curriculum for this RL phase primarily comprised the approximately 80 million problem statements generated by the auto-formalization process. This was supplemented by a small fraction (approximately 3,500 problems) of manually human-formalized problems sourced from: (1) the miniF2F-curriculum benchmark^24^; (2) the set of problems manually formalized for training the auto-formalization model; and (3) our training split of the Putnam benchmark (PutnamBench-train, derived from ref. ^21^ by excluding PutnamBench-test).

*Matchmaker system*. The matchmaker orchestrates the training process by assigning tasks to available actors. For each task, it selects a formal problem statement from the start position datasets and randomly assigns the objective as either proving or disproving the statement. Problem selection is prioritized based on ‘interestingness’, determined from the outcomes of the last *N* attempts recorded for that problem. A problem is deemed highly interesting if: (1) it has not been attempted previously; (2) it has been attempted fewer than a trust_count threshold; or (3) it has been attempted more than trust_count times but shows a mixed success rate (that is, solved in some, but not all, of the last *N* attempts). Conversely, a problem’s priority is reduced if it remains consistently unsolved after trust_count attempts, or if it has been proven for trust_count_proved consecutive attempts, indicating mastery. Statements that are successfully disproved are not retried. The simulation budget allocated to an actor for each attempt is adaptively determined: it starts with a base value and multiplicatively increases with the number of failures within the last *N* results for that specific problem up to a predefined cap value, thereby dedicating more computational resources to challenging statements. See Supplementary Table 7 for hyperparameters.

*Actor experience generation*. Each actor, upon receiving a problem statement and simulation budget from the matchmaker, interacts with the Lean environment. It performs a tree search (see ‘Prover agent’ and ‘Tree search algorithm’) guided by the current iteration of the proof network. Unlike previous work^2^, the agent never commits to one action and instead searches until it either finds a Lean-verified proof or disproof, or when its allocated simulation budget is exhausted. The verified outcome is reported back to the matchmaker, which updates its internal statistics for the problem. Proofs and disproofs discovered in the course of search are extracted and sent to the learner, failed attempts are filtered out and do not contribute to network updates.

*Learner and network updates*. The learner continuously updates the proof network parameters by training on batches of (state, tactic) pairs drawn with a fixed ratio of 10% from the Mathlib SFT dataset and 90% from a replay buffer filled with proofs and disproofs collected from the actors. The policy head is trained to predict the tactic using a cross-entropy loss. The value head is trained to predict the return obtained at the current proof (sub)goal. This iterative process of experience generation and network updates progressively enhances the proof network’s mathematical reasoning capabilities. See Supplementary Table 6 for hyperparameters.

*Computational budget summary*. The AlphaProof-specific training pipeline, which follows a general pretraining phase, involves several computationally intensive stages. The SFT stage on the Mathlib dataset was comparatively inexpensive (approximately 10 TPU days). In contrast, the subsequent stages represented a substantial investment. The auto-formalization process, which generated the approximately 80 million problem curriculum over the course of the project, required approximately 100,000 TPU days in total (equivalent to, for instance, 10 runs of 2,000 TPUs utilized for 5 days). The main RL training loop, which learned from this curriculum, was of a similar scale, spanning approximately 80,000 TPU days (equivalent to, for instance, 4,000 TPUs utilized for 20 days).

### Inference

Following main RL training, AlphaProof possesses general proof-finding capabilities for a wide range of mathematical problems. At inference time, when presented with new, unseen theorems to prove, several strategies can be used to enhance its capabilities by allocating additional computational resources.

*Scaling with tree search*. The primary method for applying more computation at inference, common in the AlphaZero family of agents^2,72^, is scaling the tree search and running multiple independent search attempts. Increasing the number of simulations performed allows the agent to refine the initial tactic suggestions provided by the proof network, integrate information over more lines of deeper reasoning and explore other potential proof sequences. This enhanced search leads to stronger tactic selection and a higher probability of finding a proof. Attempting multiple independent search attempts also increases the probability of finding a proof and offers latency benefits via parallelization. The results presented (Fig. 4a and Table 1) demonstrate the efficacy of scaling tree search compute, typically measured in compute or wall-clock time, on held-out benchmark performance.

*Scaling with TTRL*. For problems that remain intractable even with extensive tree search, AlphaProof uses TTRL for deeper, problem-specific adaptation (Fig. 2b). This process involves two main stages: the generation of a problem-specific curriculum of variants and a focused RL phase on this curriculum.

*Variant generation for TTRL curriculum*. The TTRL phase commences with the generation of a problem-specific curriculum of synthetic variants for each target Lean instance *T*. This process utilizes the Gemini^22^ LLM, prompted with few-shot examples from a curated dataset of 791 (problem, variant) Lean pairs. These exemplar pairs illustrate how the LLM can generate variants by emulating problem-solving heuristics that build on classical strategies^75^ and their formal counterparts in proof engineering, such as simplification^76^, generalization^77^, lemma proposal^78^, exploring analogies^79^ and learning from existing proofs^80^. On the basis of a sampled prompting strategy, the LLM generates either individual candidate variants or sets of correlated variants (for example, problem decompositions or simulated proof steps). In addition, programmatically created variants are generated to ensure systematic coverage of local variations (for example, by systematically altering hypotheses or goals of *T*). All generated candidates are validated for syntactic correctness in Lean. To enrich this curriculum, an evolutionary refinement process iteratively expands the variant set: promising validated variants (for example, those identified by high string similarity to *T*) recursively serve as seeds for further LLM-based generation over up to *N*_evo_ iterations (where *N*_evo_ = 15 for our reported results). This iterative cycle of LLM-based and programmatic generation, validation and evolutionary refinement, followed by deduplication, yielded hundreds of thousands of unique, syntactically valid Lean variants (*V*_*T*_) for each target *T*. This comprehensive set of variants forms a localized and structured curriculum for the subsequent RL phase (see Supplementary Data 1 for procedural details and Extended Data Fig. 5 for examples of generated variants).

*Focused RL*. Following variant generation, AlphaProof executes its core AlphaZero-inspired RL loop, initializing a specialist agent from the main RL generalist model. The key distinction from the main RL phase is the training curriculum: instead of general auto-formalized statements, the TTRL agent focuses on the problem-specific set comprising the original target problem *T* and its syntactically generated variants *V*_*T*_. This framework can be concurrently applied to multiple distinct target problems by incorporating all their respective variants into a shared start position dataset for the matchmaker, allowing for simultaneous TTRL across different target problems as presented in Fig. 4b. The subsequent learning process mirrors the main RL: distributed actors, coordinated by a matchmaker (see ‘Main RL’ and Supplementary Table 7 for TTRL-specific hyperparameters), repeatedly attempt to prove or disprove these problems. Experience, in the form of (state, tactic) pairs from successful proofs or disproofs, is used to update the specialist proof network’s parameters (see ‘Main RL’ and Supplementary Table 6 for TTRL-specific hyperparameters). To optimize the computational budget, once a target problem *T* is successfully proven, the matchmaker ceases assigning new attempts for *T* and its associated variants *V*_*T*_, as the primary objective of solving *T* has been achieved. This problem-specific adaptation allows the agent to fine-tune its learned strategies and potentially discover insights crucial for the target problem.

The TTRL phase thus enables AlphaProof to dedicate substantial, targeted computational resources at inference. By allowing the strategic reapplication of its full RL machinery for problem-specific adaptation, AlphaProof unlocks the ability to solve theorems that are intractable for the fixed, generalist model using standard inference alone. Indeed, this TTRL approach—systematically generating and solving related, often simpler, problem variants to build towards a solution for a complex target problem—is inspired by a core tenet of human mathematical problem solving, as notably described by Polya^75^. The efficacy of TTRL is demonstrated by the performance gains on held-out benchmarks (Fig. 4b and Table 1).

### Evaluation

We evaluated AlphaProof on a comprehensive suite of formal mathematics benchmarks, spanning advanced high-school to elite Olympiad and university-level problems.

#### Standard benchmarks

We introduce formal-imo, a benchmark of all non-geometry historical IMO problems that were internally formalized by experts (see ‘IMO-style geometry’ for an explanation of geometry exclusion). It contains 258 problems: 107 algebra, 77 combinatorics and 74 number theory problems (based on the official subject when available, or classification by domain experts). Detailed performance per problem of AlphaProof is shown in Extended Data Fig. 6. AlphaProof was also evaluated on our internally corrected version of the miniF2F benchmark^20^, using its ‘test’ and ‘valid’ splits as held-out test sets. These corrections addressed various misformalized problems (for example, disprovable statements, or statements with contradictions in its hypothesis) in the original dataset. AlphaProof was also evaluated on the Putnam benchmark^21^ consisting of challenging undergraduate problems. Our held-out test set, PutnamBench-test, comprised 189 problems from even-numbered years (1990 onwards). PutnamBench assigns to each one or more subjects. The most frequent ones in PutnamBench-test were algebra (78), analysis (57), number theory (33), geometry (20) and linear algebra (18). During our work, several misformalizations in the original PutnamBench were identified and subsequently corrected upstream.

#### Data separation and generalization

To ensure a rigorous evaluation of AlphaProof’s generalization capabilities, we carefully managed data separation across its training stages. Although the initial pretraining of the underlying language model utilized a broad web corpus that might have incidentally contained natural-language discussions or solutions to some historical problems, our pretraining pipeline explicitly excluded all Lean code—and critically, the formal proofs corresponding to our evaluation benchmarks—from this pretraining data. Furthermore, we only trained during the SFT phase on Mathlib, which does not contain the benchmark problems used for evaluation. The main RL training phase exclusively used the curriculum generated by our auto-formalization process and the supplementary human-formalized problems (see ‘Main RL’). Crucially, we explicitly removed all documents from the auto-formalization pipeline that were deemed too similar to any problem in the evaluation benchmarks. This data separation protocol was designed so that AlphaProof’s performance would reflect learned reasoning capabilities rather than memorization.

#### IMO-style geometry

Handling IMO-style planar geometry within Lean for AlphaProof presented challenges owing to specific gaps existing at the time of development in Mathlib’s higher-level geometry library (for example, incircles, congruence), making it impractical to even state many questions. Consequently, for dedicated Olympiad geometry problems (such as IMO 2024 P4), we used AlphaGeometry 2^3^, a system specifically designed for modern olympiad geometry. AlphaProof tackled IMO problems with mixed geometric–combinatorial aspects and all PutnamBench problems, where purely planar geometry is rare, using Mathlib’s available geometry.

#### IMO 2024 evaluation protocol and methods

For the IMO 2024 competition, a specific, predefined evaluation protocol was followed to ensure a rigorous assessment of live, out-of-distribution generalization. As part of this, an agreement was established with Prof Sir Timothy Gowers and Dr Joseph Myers to serve as independent expert judges for any solutions produced by our AI system, evaluating them according to official IMO point-awarding rules.

*Pre-competition freeze*. The main RL training of AlphaProof was halted, and all system hyperparameters were frozen before the official release of the IMO 2024 problems.

*Problem formalization*. Immediately upon release, the five non-geometry IMO 2024 problems were manually formalized into Lean by domain experts. This approach was adopted following consultation with the designated IMO judges, who indicated that the primary evaluation concern was the system’s ability to solve the formally stated problems, rather than its capacity for natural-language translation of the problem statements under competition conditions. This expert formalization also mirrors the scenario where human contestants may receive problems translated into their native language if needed.

Notably, P5 (combinatorics) proved exceptionally challenging to formalize, with initial attempts by experts being unsuccessful within the first day. Alongside this manual effort, we conducted a side experiment to test our auto-formalization system, which in fact successfully formalized P1, P2 and P6.

For problems requiring an answer (for example, ‘Find all…’), placeholders (sorry) were used for the answer component in the formalization and the system was responsible for generating candidate answers (see main text).

*Answer guessing*. Answer generation was specifically optimized to maximize the likelihood of including the correct answer within a large set of proposals (that is, optimized for pass@*k* where *k* was large), even at the cost of generating many plausible but incorrect candidates. We generated *k* = 500 answers, successfully guessing the correct answers for all applicable problems. AlphaProof was then crucial for efficiently sifting through these numerous proposals; in a low-compute refutation mode (within a 10-minute tree search per candidate), it successfully disproved 99% of incorrect Gemini-generated candidates for P1, 98% for P2 and 7% for P6, thereby isolating promising candidates for the subsequent full proof attempts.

*Geometry problem*. For reasons described in ‘IMO-style geometry’, the geometry problem (IMO 2024 P4) was addressed by AlphaGeometry 2^3^.

Verification and judging. Solutions generated by AlphaProof (P1, P2 and P6, shown in Extended Data Figs. 7–9) were first verified for correctness by the Lean kernel. Subsequently, these formal proofs were judged according to the official IMO point-awarding rules by Prof Sir Timothy Gowers and Dr Joseph Myers, who awarded full points for each solved problem.

## Online content

Any methods, additional references, Nature Portfolio reporting summaries, source data, extended data, supplementary information, acknowledgements, peer review information; details of author contributions and competing interests; and statements of data and code availability are available at 10.1038/s41586-025-09833-y.

## Supplementary information

**Extended Data Fig. 4:**
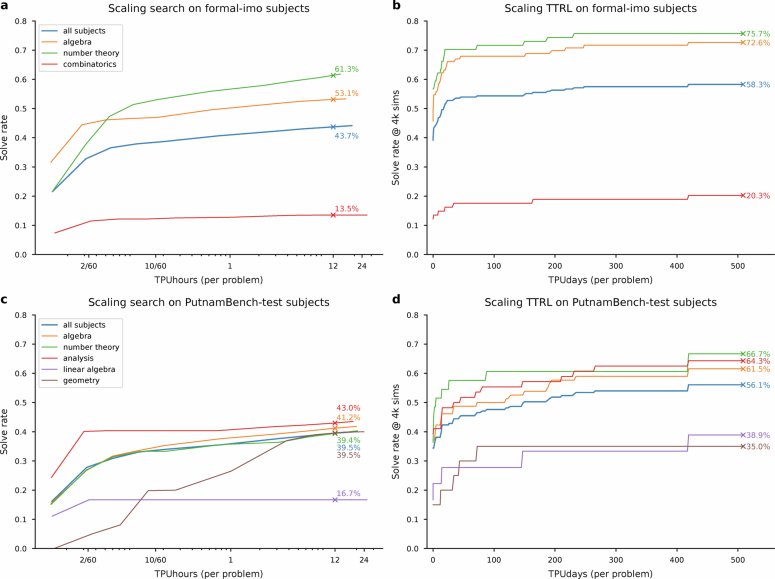
AlphaProof inference scaling dynamics by mathematical subject on formal-imo and PutnamBench-test benchmarks. Scaling of solve rates with tree search compute and TTRL on formal-imo and PutnamBench-test, broken down by subject. The thick blue line represents the overall benchmark performance, while thinner lines show performance on specific subjects.

**Extended Data Fig. 5:**
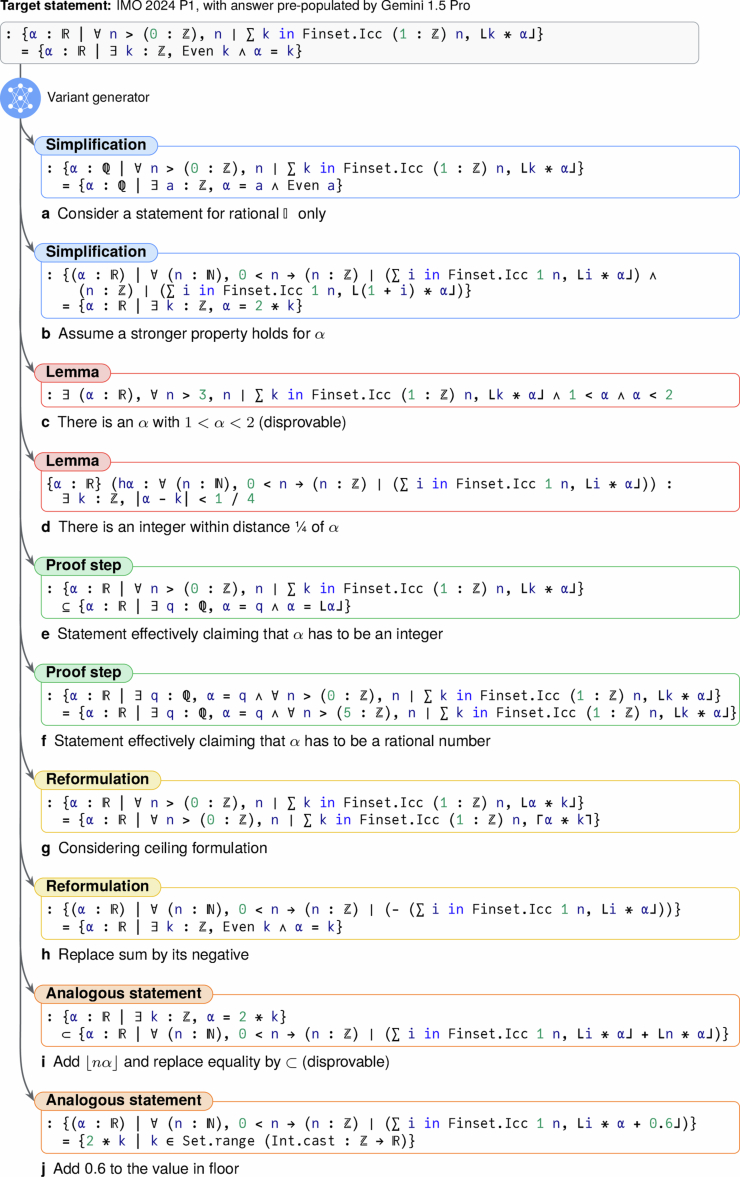
Example synthetic variant problems generated by AlphaProof automatically for the IMO 2024 P1 problem. These Lean variants, created automatically during the TTRL phase, are presented here with labels and descriptions by the authors to illustrate diverse problem-solving heuristics such as simplification, lemma proposal, proof steps, reformulation, and exploring analogies. Engaging with such variants provides insights into the target statement and facilitates problem-specific adaptation of the proof agent.

**Extended Data Fig. 6:**
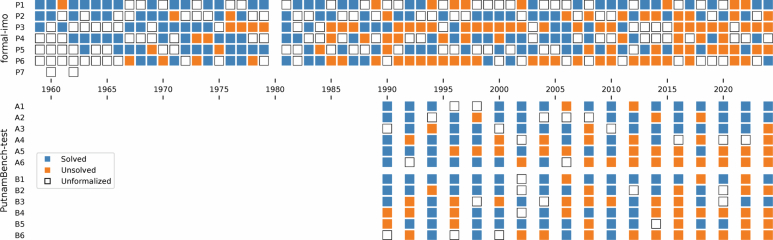
Problems solved either at the end of the TTRL phase or by main RL on the formal-imo and PutnamBench-test benchmarks. In blue, problems solved, in orange problems not solved and in white problems not formalized. The IMO competition was not held in 1980 for political reasons.

**Extended Data Fig. 7:**
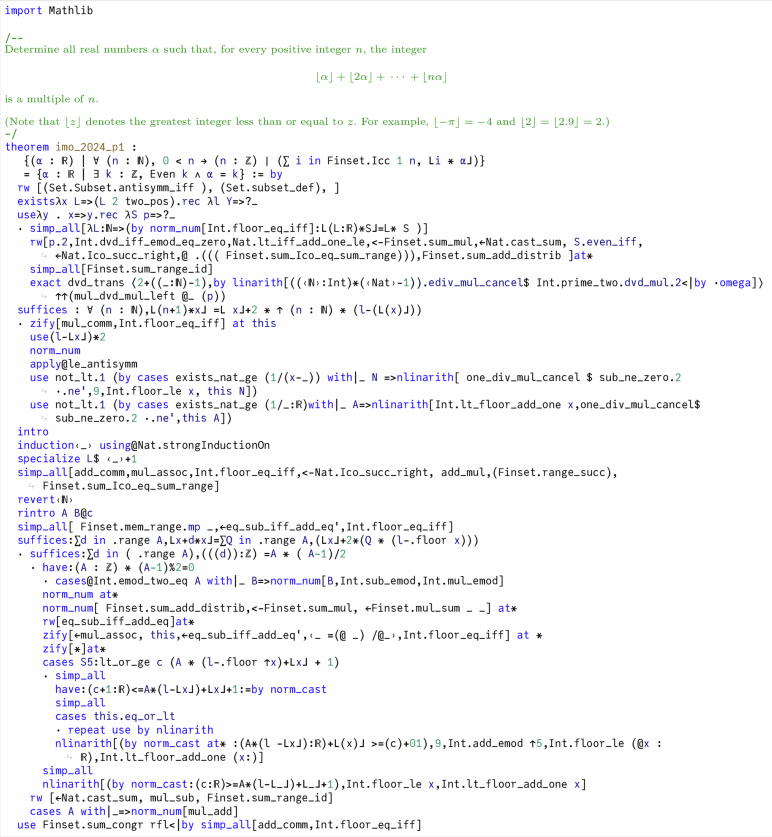
A complete proof of IMO 2024 P1 found by AlphaProof. This was found following the protocol in section 6.4. An interactive version of this figure (along with Extended Data Fig. 8 and Extended Data Fig. 9), which allows inspecting all the intermediate Lean goal states is available in^28^. This algebra problem was fully solved by 413 of the 609 student contestants^27^. Figure adapted from ref. ^28^.

**Extended Data Fig. 8:**
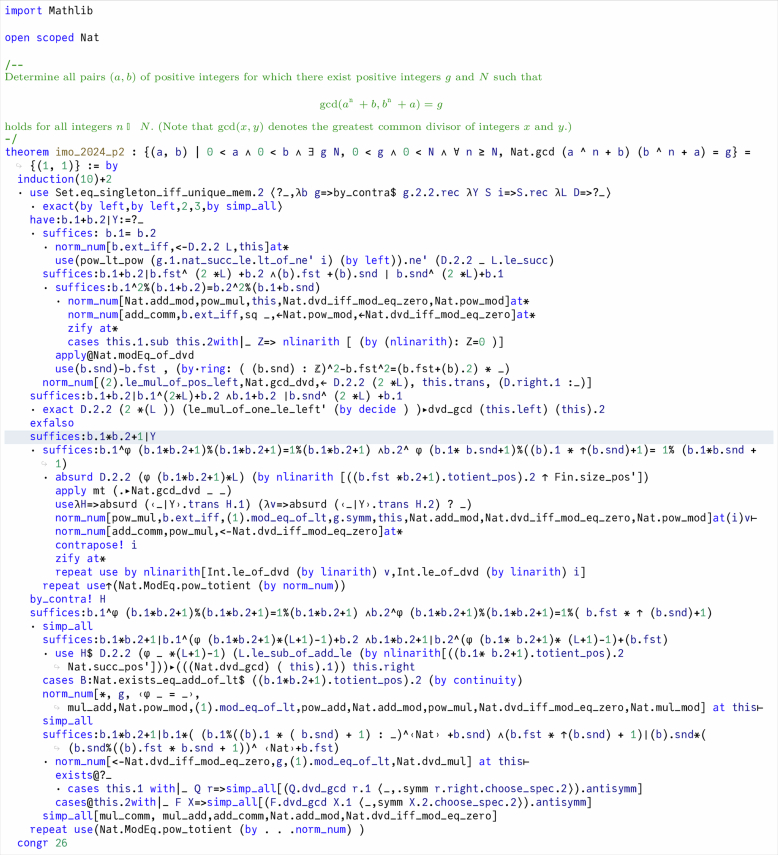
A complete proof of IMO 2024 P2 found by AlphaProof. This was found following the protocol in section 6.4. The crux of this proof is considering the properties of *ab* + 1 (e.g. that it divides *g*), which can be seen on the highlighted line. This number theory problem was fully solved by 156 of the 609 student contestants^27^. Figure adapted from ref. ^28^.

**Extended Data Fig. 9:**
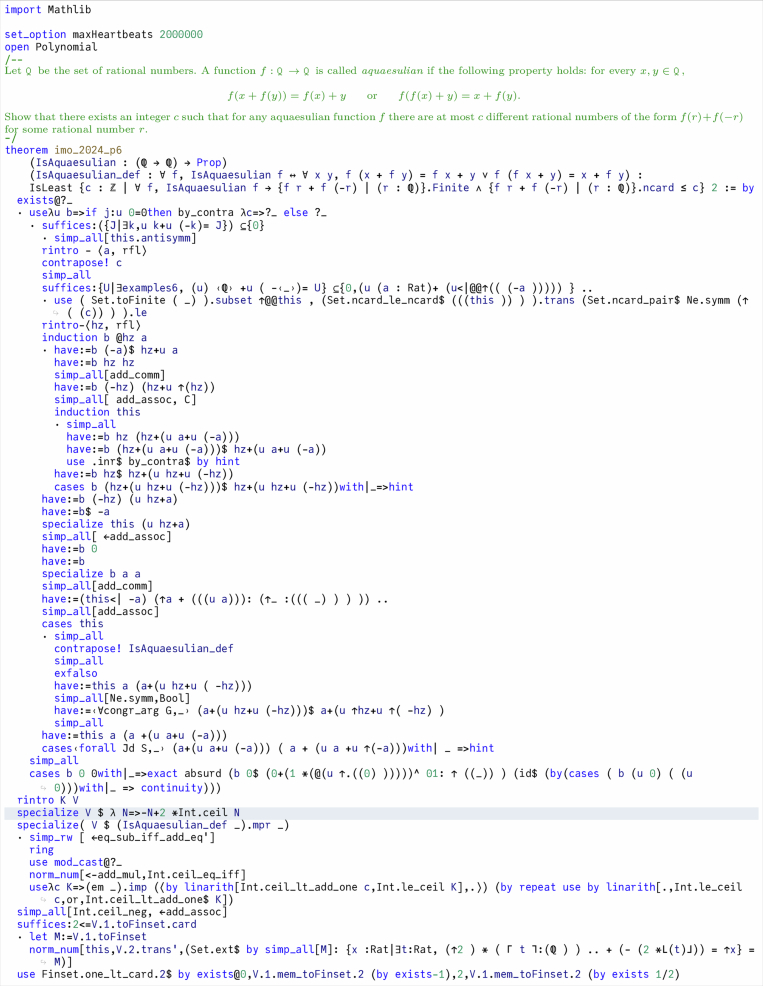
A complete proof of IMO 2024 P6 found by AlphaProof. This was found following the protocol in section 6.4. The highlighted line shows the key insight of constructing the function *f*(*x*) = − *x* + 2⌈*x*⌉. This algebra problem was fully solved by only 5 of the 609 student contestants^27^. Figure adapted from ref. ^28^.

Supplementary Information This PDF contains numerical solve rates supplementing Extended Data Fig. 4, hyper-parameter values, details on problems used to benchmark auto-formalization and sample proofs from the AlphaProof agent. These proofs are primarily from the PutnamBench benchmark. The file includes Supplementary Tables 1–7 and Figs. 1–6.

Supplementary Data 1 Pseudocode written in Python elaborating the high-level structure of AlphaProof: RL, auto-formalization and variant generation.

## Figures and Tables

**Extended Data Table 1:**
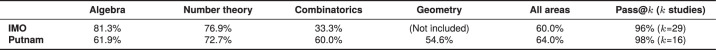
Performance of the auto-formalization process on internal benchmarks of fifty representative IMO and Putnam problems

The exact problems constituting these benchmarks are listed in Supplemental Table 5. All numbers are pass@1, except the last column which gives the proportion of problems correctly auto-formalized in any one of our *k* studies, as rated by Lean experts.

**Extended Data Table 2:**
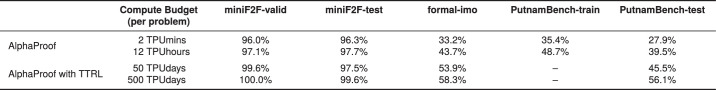
Detailed performance of AlphaProof configurations across all benchmarks

The “compute budget (per problem)” refers to the average computational cost as defined in Fig. 4a. 100% of the miniF2F^20^ valid split was proved. We observe similar scaling properties of tree search on PutnamBench-train and PutnamBench-test^21^. TTRL was not run on the PutnamBench-train set.

## Data Availability

The human-authored formal proofs used for supervised fine-tuning were drawn from the publicly available Mathlib library^4^. Our corrected versions of the miniF2F benchmark and the formal-imo benchmark are available at https://github.com/google-deepmind/miniF2F and https://github.com/google-deepmind/formal-imo, respectively. The Putnam benchmark problems come from commit https://github.com/trishullab/PutnamBench/tree/02b2cff66d7ac8e9372c778c4ab9ec80082ecbd8 of the publicly available PutnamBench^21^. The formal problem statements that form the primary curriculum for the main RL phase, as well as the problem variants generated for TTRL, were synthetically produced by AlphaProof’s auto-formalization and variant generation components, respectively, as described in Methods (‘Auto-formalization’ and ‘Scaling with TTRL’). Similarly, the proofs used to train AlphaProof during RL were generated by the system itself through interaction with the Lean environment as described in Methods (‘Lean environment’ and ‘Main RL’).

## Code availability

The pseudocode for the AlphaProof algorithm can be found in the file alphaproof_pseudocode.py in Supplementary Data 1. Detailed hyperparameters for the training and search procedures are described throughout Methods and summarized in the Supplementary tables. The Lean 4 proof assistant, which forms the interactive environment for AlphaProof, is open source and available at https://lean-lang.org/. The Mathlib library, used extensively by AlphaProof, is also an open-source community project, accessible at https://github.com/leanprover-community/mathlib4. We are making AlphaProof’s capabilities available to members of the scientific community through an interactive tool communicating with Google servers. Researchers interested in gaining access will be able to apply at https://deepmind.google/alphaproof.
