# Supplementary material for: Olympiad-level formal mathematical reasoning with reinforcement learning
Source: Nature. 2025 Nov 12;651(8106):607–13. doi: 10.1038/s41586-025-09833-y (PMC12999475; doi:10.1038/s41586-025-09833-y)
Supplement: Supplementary file 1 — This PDF contains numerical solve rates supplementing Extended Data Fig. 4, hyper-parameter values, details on problems used to benchmark auto-formalization and sample proofs from the AlphaProof agent. These proofs are primarily from the PutnamBench benchmark. The file includes Supplementary Tables 1–7 and Figs. 1–6. [file 41586_2025_9833_MOESM1_ESM.pdf]

nature portfolio

https://doi.org/10.1038/s41586-025-09833-y

# Supplementary information

# Olympiad-level formal mathematical reasoning with reinforcement learning

In the format provided by the authors and unedited

# Supplementary Information for: Olympiad-level formal mathematical reasoning with reinforcement learning

Thomas Hubert [*], Rishi Mehta, Laurent Sartran, Miklós Z. Horváth, Goran Žužić, Eric Wieser [†], Aja Huang, Julian Schrittwieser, Yannick Schroecker, Hussain Masoom, Ottavia Bertolli, Tom Zahavy, Amol Mandhane, Jessica Yung, Iuliya Beloshapka, Borja Ibarz, Vivek Veeriah, Lei Yu, Oliver Nash, Paul Lezeau, Salvatore Mercuri, Calle Sönne, Bhavik Mehta, Alex Davies, Daniel Zheng, Fabian Pedregosa, Yin Li, Ingrid von Glehn, Mark Rowland, Samuel Albanie, Ameya Velingker, Simon Schmitt, Edward Lockhart, Edward Hughes, Henryk Michalewski, Nicolas Sonnerat, Demis Hassabis, Pushmeet Kohli [‡] and David Silver

*Google DeepMind, London, United Kingdom*

*Google DeepMind*

| Agent | Compute Budget (per problem) | formal-imo | | | putnam-test [1] | | | | |
|---|---|---|---|---|---|---|---|---|---|
| | | algebra | combina-torics | number-theory | algebra | analysis | geometry | linear-algebra | number-theory |
| AlphaProof | 2 TPUmins | 44.8% | 11.2% | 38.6% | 26.9% | 40.1% | 4.3% | 16.5% | 27.2% |
| | 12 TPUhours | 53.1% | 13.5% | 61.3% | 41.2% | 43.0% | 39.5% | 16.7% | 39.5% |
| AlphaProof with TTRL | 50 TPUdays | 67.9% | 17.6% | 70.3% | 48.7% | 51.8% | 30.0% | 27.8% | 57.6% |
| | 500 TPUdays | 72.6% | 20.3% | 75.7% | 61.5% | 64.3% | 35.0% | 38.9% | 66.7% |

Supplementary Table 1: **AlphaProof solve rates by mathematical subject at key inference compute budgets.** Performance of AlphaProof on the formal-IMO and putnam-test benchmarks, disaggregated by mathematical subject, at selected compute budgets for tree search (TPUminutes, TPUhours) and TTRL (TPUdays).

| Hyperparameter | Value |
|---|---|
| Total Parameters | 3B |
| Encoder Blocks | 18 |
| Decoder Blocks | 24 |
| Attention Heads | 16 |
| Attention Head Size | 128 |
| Feed Forward Widening Factor | 6 |
| Policy: number of parallel tactics | 6 |
| Value: number of bins | 64 |

Supplementary Table 2: Prover network architecture hyperparameters.

[*] tkhubert@google.com
[†] efw@google.com
[‡] pushmeet@google.com

| Hyperparameter | Value |
|---|---|
| Num sampled tactics $K$ | 6 |
| Progressive sampling $C$ | 0.01 |
| Progressive sampling $\alpha$ | 0.6 |
| UCB value discount $\beta$ | 0.99 |
| UCB prior temperature $\tau$ | 200 |
| UCB exploration constant $c_{\text{init}}$ | 0.001 |
| UCB exploration constant $c_{\text{base}}$ | 3200 |
| UCB AND-node prior multiplier $c_{\text{AND}}$ | 64 |
| UCB unvisited children value penalty | 32 |

Supplementary Table 3: Search hyperparameters.

| | Pre-Training | Mathlib SFT |
|---|---|---|
| Dataset Size | 300B tokens | 300k state-tactic pairs |
| Batch Size | 4096 | 4096 |
| Training Steps | 3M | 500 |
| Encoder Size | 1024 tokens | – |
| Decoder Size | 256 tokens | 64 tokens |

Supplementary Table 4: Training hyperparameters used during Pre-training, Supervised Finetuning and RL.

| IMO | |
|---|---|
| imo_2000_p2 | imo_2000_p3 |
| imo_2000_p4 | imo_2000_p5 |
| imo_2002_p1 | imo_2002_p3 |
| imo_2002_p4 | imo_2002_p5 |
| imo_2004_p2 | imo_2004_p3 |
| imo_2004_p4 | imo_2004_p6 |
| imo_2006_p2 | imo_2006_p3 |
| imo_2006_p4 | imo_2006_p5 |
| imo_2008_p2 | imo_2008_p3 |
| imo_2008_p4 | imo_2008_p5 |
| imo_2010_p1 | imo_2010_p3 |
| imo_2010_p5 | imo_2010_p6 |
| imo_2012_p2 | imo_2012_p3 |
| imo_2012_p4 | imo_2012_p6 |
| imo_2014_p1 | imo_2014_p2 |
| imo_2014_p5 | imo_2014_p6 |
| imo_2016_p2 | imo_2016_p3 |
| imo_2016_p4 | imo_2016_p5 |
| imo_2016_p6 | imo_2018_p2 |
| imo_2018_p3 | imo_2018_p4 |
| imo_2018_p5 | imo_2020_p2 |
| imo_2020_p3 | imo_2020_p4 |
| imo_2020_p5 | imo_2022_p1 |
| imo_2022_p2 | imo_2022_p3 |
| imo_2022_p5 | imo_2022_p6 |

| Putnam | |
|---|---|
| putnam_1962_a5 | putnam_1964_a2 |
| putnam_1964_a5 | putnam_1964_b1 |
| putnam_1964_b6 | putnam_1968_b5 |
| putnam_1970_b2 | putnam_1974_a1 |
| putnam_1974_b3 | putnam_1974_b6 |
| putnam_1984_a3 | putnam_1984_b2 |
| putnam_1988_b1 | putnam_1990_b2 |
| putnam_1994_a5 | putnam_1994_b2 |
| putnam_1994_b3 | putnam_1994_b4 |
| putnam_1996_a2 | putnam_1996_a3 |
| putnam_1998_b2 | putnam_2000_a2 |
| putnam_2002_a2 | putnam_2002_b6 |
| putnam_2004_a1 | putnam_2004_a4 |
| putnam_2004_a5 | putnam_2004_b4 |
| putnam_2006_a1 | putnam_2006_b3 |
| putnam_2006_b5 | putnam_2008_a5 |
| putnam_2008_b3 | putnam_2008_b6 |
| putnam_2010_a5 | putnam_2010_b3 |
| putnam_2012_a2 | putnam_2012_b3 |
| putnam_2014_a1 | putnam_2016_b4 |
| putnam_2016_b6 | putnam_2018_a4 |
| putnam_2018_a5 | putnam_2018_b5 |
| putnam_2020_a1 | putnam_2020_b1 |
| putnam_2022_a1 | putnam_2022_a6 |
| putnam_2022_b4 | putnam_2022_b5 |

Supplementary Table 5: The fifty IMO and Putnam problems used for internal benchmarking of auto-formalization.

| | Main RL | TTRL |
|---|---|---|
| Fraction SFT | 10% | 25% |
| SFT Dataset | 300k mathlib state-tactic pairs | 300k mathlib state-tactic pairs and Main RL model samples |
| RL Replay Buffer Size | 60M state-tactic pairs | 20M state-tactic pairs |
| Batch Size | 4096 | – |
| Policy Loss Weight | 1.0 | – |
| Value Loss Weight | 1e-3 | – |
| Training Steps | 1M | 2.5M |

Supplementary Table 6: Learner hyperparameters used during RL.

| | MainRL | TTRL |
|---|---|---|
| Disprove rate | 50% | – |
| trust_count | 8 | 5 |
| trust_count_proved | 12 | – |
| Weight interesting | 1.0 | – |
| Weight undecided | 0.1 | – |
| Weight fully proved | 0.001 | – |
| Weight disproved | 0.0 | – |
| Start number simulations | 250 | 125 |
| Multiplicative factor for each failure | 1.17 | 2 |
| Cap number simulations | 16000 | – |

Supplementary Table 7: Matchmaker hyperparameters used during RL.

# Proofs found by AlphaProof

```
import Mathlib

/--
The real numbers a, b, c, d are such that a ≥ b ≥ c ≥ d > 0 and a + b + c + d = 1. Prove that

                    (a + 2b + 3c + 4d)a^a b^b c^c d^d < 1.
-/
theorem imo_2020_p2 (a b c d : ℝ) (hba : b ≤ a) (hcb : c ≤ b) (hdc : d ≤ c)
    (hd : 0 < d) (habcd : a + b + c + d = 1) :
    (a + 2 * b + 3 * c + 4 * d) * a ^ a * b ^ b * c ^ c * d ^ d < 1 := by
  have' := Real.geom_mean_le_arith_mean4_weighted (by linarith : 0 ≤ a) (by linarith : 0 ≤ b) (by linarith : 0 ≤ c) hd.le
    ↪ (by linarith : 0 ≤ a) (by linarith : 0 ≤ b)
  nlinarith [this (hd.le.trans hdc) hd.le habcd,pow_three (a-b),pow_three (b-c),pow_three (c-d),pow_three (1-a)]
```

Supplementary Figure 1: A proof found by AlphaProof for an algebra IMO problem (P2 2020).

```
import Mathlib

theorem putnam_2000_a1 (A : ℝ) (Apos : A > 0) :
    ({S : ℝ | ∃ x : ℕ → ℝ, (∀ j : ℕ, x j > 0) ∧ (∑' j : ℕ, x j) = A ∧ (∑' j : ℕ, (x j) ^ 2) = S}
      = ((fun A : ℝ => Set.Ioo 0 (A ^ 2)) : ℝ → Set ℝ) A) := by
  apply@Set.ext
  aesop
  · have:=not_imp_comm.1 tsum_eq_zero_of_not_summable (Apos.ne')
    exact (le_tsum (((this.mul_right @_).of_nonneg_of_le (by((bound)))) fun and' =>sq ↑( w _)▸ (mul_le_mul_left <| left
      ↪ _).mpr ↑(le_tsum this @_ fun and from' =>le_of_lt (( left (and))))) 10 (by ((bound)))).trans_lt' (by {bound})
  · by_cases h:Summable w∧Summable (w ·^2)
    · rw[sq,←tsum_mul_right,←sub_pos,←tsum_sub (h.1.mul_right _) h.2]
      exact (le_tsum.comp (h.1.mul_right _).sub h.2 0 (by (norm_num [le_tsum _,le_of_lt _, left, true,h, mul_le_mul_left
        ↪ _,sq]))).trans_lt' (by. (norm_num [ ↑(sum_le_tsum {0,1} ↑_ h.left).trans_lt',le_of_lt _,sq, false, left]))
    apply (tsum_eq_zero_of_not_summable (h ∘.intro (by_contradiction (Apos.ne.symm
      ↪ ∘(tsum_eq_zero_of_not_summable)))))▸pow_pos Apos (2)
  by_cases h:∃a,0<a∧∑'b,(a^b*(A/∑'b,a^b))^2=x
  · exact h.elim fun and ⟨a, H⟩=> if I:_ then by aesop else⟨ _, fun and=>mul_pos (pow_pos a and) (div_pos Apos
    ↪ ((tsum_nonneg (by bound)).lt_of_ne' I)),tsum_mul_right.trans (mul_div_cancel₀ A I),H⟩
  field_simp[sq,mul_assoc,mul_comm,mul_left_comm,<-mul_pow,tsum_div_const,tsum_mul_left] at h
  by_cases h:∃ a ∈Set.Ioo @0 1, A*(A *(1-a * a)⁻¹) =x * ( (1-a)⁻¹* (1-a)⁻¹)
  · cases h.elim fun and⟨ ⟨a, _⟩, _⟩=>‹∀ (x _),¬_› and a (by rw [tsum_geometric_of_lt_one (mul_self_nonneg _) (by
    ↪ nlinarith),tsum_geometric_of_lt_one a.le (by valid), ( (by assumption:)),div_eq_iff (ne_of_gt (by(bound)))])
  field_simp[←geom_sum_mul_neg] at h
  field_simp+contextual[show(1-_*_: ℝ)=(1- _)*(_+1)by ring,←div_div, sub_ne_zero.2 ∘ne_of_gt] at h
  rcases ((intermediate_value_Ioo') zero_le_one (by(((fun_prop)))) (by repeat use (by linarith))).elim fun and=>And.elim
    ↪ <| sub_ne_zero.mpr ∘ And.elim (h _)
```

Supplementary Figure 2: A proof found by AlphaProof for an analysis problem involving infinite sums. This is `putnam_2000_a1` from PutnamBench.

```lean
import Mathlib

open Metric Classical

theorem putnam_1996_a2 (O1 O2 : EuclideanSpace ℝ (Fin 2)) (C1 C2 : Set (EuclideanSpace ℝ (Fin 2)))
    (hC1 : C1 = sphere O1 1) (hC2 : C2 = sphere O2 3) (hO1O2 : dist O1 O2 = 10) :
    {M : EuclideanSpace ℝ (Fin 2) | ∃ X Y, X ∈ C1 ∧ Y ∈ C2 ∧ M = midpoint ℝ X Y}
      = ((fun O1 O2 : EuclideanSpace ℝ (Fin 2) => {p : EuclideanSpace ℝ (Fin 2) | dist p (midpoint ℝ O1 O2) ≥ 1
        ∧ dist p (midpoint ℝ O1 O2) ≤ 2}) : (EuclideanSpace ℝ (Fin 2)) → (EuclideanSpace ℝ (Fin 2)) → Set (EuclideanSpace
        ↪ ℝ (Fin 2))) O1 O2 := by
  use hC1▸hC2▸le_antisymm ( fun and⟨x,y,A, B, _⟩=>⟨? _,?_⟩) fun and ⟨a, _⟩=> if a:IsPreconnected (Metric.sphere O1 1)
    ↪ then(? _)else(? _)
  · rewrite [@isPreconnected_closed_iff] at a
    specialize (a {b |dist (2 • and-b) (O2) ≤3} {C | dist (2 •and -C) O2≥3}) ↑(isClosed_le ↑( (by fun_prop) )
      ↪ continuous_const) (isClosed_le continuous_const (by(((fun_prop))))) _ _ _
    · exact (fun A B=>le_total (dist _ _) _)
    · use O1+(1/dist and (midpoint ℝ O1 (O2))) • (and-midpoint ℝ O1 (O2)),.symm (by field_simp [norm_smul_of_nonneg _,
      ↪ ←dist_eq_norm])
      field_simp[midpoint_eq_smul_add,dist_eq_norm]at‹ 1 ≤dist _ _›‹_ ≤_› ⊢
      rw[(by module:_-O2=(2-1/norm _) • (and-((1/2: ℝ) •O1+(1/2: ℝ) •O2)))]
      exact (.trans (by rw [norm_smul_of_nonneg (by linear_combination inv_le_one a)]) (by linear_combination‹_ ≤_›*2
        ↪ -div_self (one_pos.trans_le a).ne'))
    · use O1-(1/dist and (midpoint ℝ O1 (O2))) • (and-midpoint ℝ O1 (O2)),by field_simp [norm_smul_of_nonneg,
      ↪ ←dist_eq_norm]
      norm_num[midpoint_eq_smul_add,dist_eq_norm]at‹ 1 ≤dist _ _›⊢
      apply(((congr_arg _) (by module)).trans (norm_smul_of_nonneg (add_nonneg (2).cast_nonneg (inv_nonneg.2
        ↪ (zero_le_one.trans a))) ( and-((1/2:ℝ) •O1+ (1/2 : ℝ) •O2)))).ge.trans'
      linear_combination (2*a)-div_self (one_pos.trans_le a).ne'
    exact a.elim fun A B=>⟨A,_, B.1, B.2.elim le_antisymm,by simp_arith[midpoint_eq_smul_add,←Nat.cast_smul_eq_nsmul ℝ]⟩
  · convert (a ((isPreconnected_sphere _ _ _))).elim
    norm_num only[←Module.finrank_eq_rank,finrank_euclideanSpace_fin]
  · simp_rw [by assumption',Metric.mem_sphere,dist_eq_norm_vsub,midpoint_vsub_midpoint] at hO1O2 A B⊢
    simp_all[midpoint_eq_smul_add,norm_smul]
    refine not_lt.1 (mt ((norm_sub_le _ _).trans_lt ∘ (add_lt_add_left · (norm ((2 : ℝ)⁻¹ • (x-O1))))) (by
      ↪ norm_num[norm_smul_of_nonneg,A, B]))
  zify[*,dist_eq_norm_vsub,midpoint_vsub_midpoint]at A B ⊢
  norm_num[←dist_eq_norm, A.out,B.out,midpoint_eq_smul_add,norm_smul_of_nonneg,(norm_add_le _ _).trans]
```

Supplementary Figure 3: A proof found by AlphaProof for a geometry problem.
This is `putnam_1996_a2` from PutnamBench.

```
import Mathlib

open scoped Classical InnerProductSpace
open Metric

notation "ℝ³" => EuclideanSpace ℝ (Fin 3)

theorem putnam_2002_a2 (unit_sphere : Set ℝ³) (hsphere : unit_sphere = sphere 0 1)
    (hemi : ℝ³ → Set ℝ³) (hhemi : hemi = fun V ↦ {P : ℝ³ | ⟪P, V⟫_ℝ ≥ 0}) :
    ∀ (S : Set ℝ³), S ⊆ unit_sphere ∧ S.encard = 5 → ∃ V : ℝ³, V ≠ 0 ∧ (S ∩ hemi V).encard ≥ 4 := by
  use‹_›▸hsphere▸fun A B=>by_contra fun and=> if a : A.Finite then (B.2.not_lt) ?_ else(B.2.not_lt) ?_
  · lift A to Finset ℝ³ using a
    field_simp(disch:=intros)[Set.subset_def,Set.encard_eq_coe_toFinset_card]at and B⊢
    have:Matrix.det @![ (0 :ℝ³),0,0]=0
    · exact (Matrix.det_eq_zero_of_row_eq_zero 0 (by((subsingleton))))
    let α := A.equivFinOfCardEq ↑(mod_cast B.right)
    have:Matrix.det ![ (α.symm 0).val, - (α.symm (1)).val,0]=0 := by_contra fun and' =>?_
    · refine absurd (Matrix.exists_mulVec_eq_zero_iff.2 this) (fun ⟨a, R, M⟩=>not_le_of_lt (and a R) ((.trans (? _) ↑(by
      ↪ rw [ Finset.card_filter,← A.sum_coe_sort,←α.symm.sum_comp]))))
      use not_lt.1 fun and' =>not_le_of_lt (and _ (neg_ne_zero.2 R)) (.trans (?_) (by rw [ Finset.card_filter _,←
        ↪ A.sum_coe_sort,←α.symm.sum_comp]))
      norm_num[←List.ofFn_inj,Matrix.mulVec,Matrix.dotProduct, Fin.sum_univ_five] at M (and')⊢
      simp_rw [ Fin.sum_univ_three _, Finset.card_filter, Fin.sum_univ_five]at (and')⊢
      cases le_or_lt 0 ((α.symm @2).1 0 * a 0+ (α.symm (2)).val (1) * a (1)+ (α.symm (2)).val (2) * a 2)
      · cases le_or_lt 0 ((α.symm 3).val 0 * a 0+ (α.symm (3)).val (1) * a (1)+ (α.symm (3)).val (2) * a 2)
        · cases (and').not_le (by norm_num[ (by linarith:0 ≤ (α.symm (1)).val 0 * a 0+ (α.symm (1)).val 1 * a (1)+ (α.symm
          ↪ (1)).val (2) * a 2), *])
        cases le_or_lt 0 ((α.symm 4).1 0 * a 0+ (α.symm 4).1 (1) * a (1)+ (α.symm 4).1 (2) * a 2)
        · norm_num[ (by linear_combination-M.2: (α.symm (1)).val 0 * a 0+ (α.symm (1)).val (1) * a (1)+ (α.symm (1)).val
          ↪ (2) * a 2 =0),not_le_of_lt @‹_›, *]at and'
        simp_arith only[*, one_mul,le_refl, if_pos,show (α.symm 1).1 0 * a 0+ (α.symm 1).1 1 * a 1+ (α.symm 1).1 2*a 2 = 0
          ↪ by linarith,le_of_lt]
        omega
      cases le_or_lt 0 ((α.symm 3).val 0 * a 0+ (α.symm (3)).val (1) * a (1)+ (α.symm (3)).val (2) * a 2)
      · cases le_or_lt 0 ((α.symm 4).1 0 * a 0+ (α.symm 4).1 (1) * a (1)+ (α.symm 4).1 (2) * a 2)
        · norm_num[ (by (linear_combination-M.2): (α.symm (1)).val 0 * a 0 + (α.symm (1)).val (1) * a (1)+ (α.symm
          ↪ (1)).val (2) * a 2 =0),not_le_of_lt (by assumption), *]at and'
        exact (congr_arg₂ _ (by rw [if_pos M.1.le, if_pos (by linarith),if_pos (by gcongr)]) (if_pos (by
          ↪ gcongr))).ge.trans' (by omega)
      push_cast[*, if_pos, false,refl, true, (by(linear_combination-M.2): (α.symm (1)).val 0 * a 0+ (α.symm (1)).val (1) *
        ↪ a (1)+ (α.symm (1)).val (2) * a 2 =0),le_self_add]
      push_cast[*,le_self_add,le_of_lt]
    exact (and') ((Matrix.det_eq_zero_of_row_eq_zero (2) (by(subsingleton))))
  rcases B.right▸ A.encard_eq_top a
```

Supplementary Figure 4: A proof found by AlphaProof for a combinatorics problem with a geometric flavour. This is `putnam_2002_a2` from PutnamBench.

```
import Mathlib

open Real

theorem putnam_2016_a3 (f : ℝ → ℝ) (hf : ∀ x : ℝ, x ≠ 0 → f x + f (1 - 1 / x) = arctan x) :
    (∫ x in (0)..1, f x = (3 * Real.pi / 8 : ℝ)) := by
  inhabit Real
  trans∫R in(0)..1,(R.arctan-(1-1/R).arctan+(1-1/(1-1/R)).arctan) / 2
  · apply intervalIntegral.integral_congr_ae.comp ( ((Set.countable_singleton (0 ) ).insert @1).ae_not_mem _).mono
    simp_arith+contextual[←hf, sub_eq_zero,inv_neg,add_sub_add_comm]
    field_simp+contextual [← add_assoc, sub_ne_zero, true,div_neg]
  rw[intervalIntegral.integral_div,intervalIntegral.integral_of_le (by
   ↪ norm_num),MeasureTheory.integral_Ioc_eq_integral_Ioo,MeasureTheory.setIntegral_congr measurableSet_Ioo fun and m
   ↪ =>by rw [one_sub_div m.1.ne']]
  rcases ↑(isEmpty_or_nonempty ℝ)
  · subsingleton
  rw[MeasureTheory.setIntegral_congr measurableSet_Ioo fun and (B) =>by rw [ one_div_div, one_sub_div ↑( sub_ne_zero.mpr
   ↪ (B.right).ne)]]
  by_cases h:MeasureTheory.IntegrableOn (fun A=> A.arctan-Real.arctan ((A-1)/A)) (.Ioo 0 1)
  · by_cases h:MeasureTheory.IntegrableOn ( fun and=>(-1/ (and-1):Real).arctan) (.Ioo ↑0 1)
    · simp_all[←intervalIntegrable_iff_integrableOn_Ioo_of_le]
      simp_all[←intervalIntegral.integral_of_le _,MeasureTheory.integral_Ioc_eq_integral_Ioo.symm,
       ↪ sub_add_eq_add_sub,intervalIntegral.integral_comp_sub_right Real.arctan,neg_div,div_eq_iff]
      by_cases h:IntervalIntegrable (fun p=>((p-1)/p).arctan) MeasureTheory.volume 0 (1)
      · simp_all[intervalIntegral,MeasureTheory.integral_Ioc_eq_integral_Ioo]
        rw [←MeasureTheory.setIntegral_congr measurableSet_Ioo fun and c=>by rw [Real.arctan_inv_of_neg (sub_neg.2 c.2)]]
        norm_num[←intervalIntegral.integral_of_le
         ↪ _,MeasureTheory.integral_Ioc_eq_integral_Ioo.symm,Continuous.intervalIntegrable (by apply
         ↪ Real.continuous_arctan.comp (by fun_prop):Continuous fun and=>
         ↪ (and-1).arctan),Real.continuous_arctan.intervalIntegrable]
        norm_num[h,Continuous.intervalIntegrable (by apply Real.continuous_arctan.comp (by fun_prop):Continuous fun and=>
         ↪ (and-1).arctan),Real.continuous_arctan.intervalIntegrable]
        replace hf:∫x in(0)..1,((x-1)/x).arctan=∫x in(0)..1,-(π/4) :=(sub_zero (1: ℝ)▸sub_self (1: ℝ)▸.trans
         ↪ (intervalIntegral.integral_comp_sub_left _ _).symm ? _)
        · exact (.trans (by rw [←add_assoc _, (hf), ←neg_zero,← intervalIntegral.integral_comp_neg]) ↑(.trans ( by aesop)
         ↪ (by ring)))
        rw [←sub_eq_zero, sub_sub_self,←intervalIntegral.integral_sub _ (by bound)]
        · rw [←neg_eq_self ℕ,sub_self,<-intervalIntegral.integral_neg,←sub_zero 1,←sub_self
         ↪ 1,←intervalIntegral.integral_comp_sub_left,eq_comm]
          norm_num[intervalIntegral,MeasureTheory.integral_Ioc_eq_integral_Ioo, sub_div,neg_div]
          refine MeasureTheory.setIntegral_congr measurableSet_Ioo fun and ⟨a, _⟩=>?_
          field_simp
          exact (symm ((.trans (by rw [←neg_sub 1,neg_div,Real.arctan_neg]) (by linarith[inv_div and (1 -
           ↪ (and))▸Real.arctan_inv_of_pos (by bound)]))))
        norm_num[(h.comp_sub_left 1).mono_set _ |>.congr ↑(.of_forall _),sub_div]
      simp_all[(Real.continuous_arctan.intervalIntegrable _ _).sub (by gcongr) |>.congr (.of_forall _),]
    cases h ((MeasureTheory.integrable_const _).mono' (measurable_const.mul (by
     ↪ fun_prop)).arctan.aestronglyMeasurable<|.of_forall fun and=>abs_le.2<|Set.mem_Icc_of_Ioo<|Real.arctan_mem_Ioo _)
  absurd h ∘(Real.continuous_arctan.integrableOn_Icc.mono_set Set.Ioo_subset_Icc_self).sub
  exact (MeasureTheory.integrable_const _).mono' ((measurable_sub_const _).mul measurable_inv).arctan.aestronglyMeasurable
   ↪ (.of_forall fun and=>abs_le.2 (Set.mem_Icc_of_Ioo (Real.arctan_mem_Ioo _) ) )
```

Supplementary Figure 5: A proof found by AlphaProof for an analysis problem about calculating an integral. This is `putnam_2016_a3` from PutnamBench.

```
import Mathlib

open Function Polynomial

theorem putnam_2018_b5 (f : (Fin 2 → ℝ) → (Fin 2 → ℝ)) (h₁ : ContDiff ℝ 1 f)
    (h₂ : ∀ x i j, 0 < fderiv ℝ f x (Pi.single i 1) j)
    (h₃ : ∀ x, 0 < fderiv ℝ f x ![1, 0] 0 * fderiv ℝ f x ![0, 1] 1 - (1 / 4) * (fderiv ℝ f x ![1, 0] 1 + fderiv ℝ f x ![0,
      ↪ 1] 0) ^ 2) : Injective f := by
  refine'λa B b=>by_contra (λ (x :) => _)
  let R M:= f$ AffineMap.lineMap B a (M: ℝ)
  suffices(M):deriv R M 0 * ( a 0-B 0)+deriv R M (1) * ( a (1)-B (1)) >(0)
  · suffices:StrictMonoOn (λM=> R M 0*(a 0-B 0)+ R M (1) * ( a (1)-B (1))) (.Icc ↑0 1)
    · use (by norm_num[b,R]:¬ _)$ this unitInterval.zero_mem unitInterval.one_mem
    suffices(M):DifferentiableAt ℝ R M
    · apply ((strictMono_of_deriv_pos) (‹∀ (x : ℝ),_ <_› ·|>.trans_eq (by ·norm_num [differentiableAt_pi.1 ↑((this)
      ↪ _),deriv_pi, $(id)deriv_mul_const]))).strictMonoOn
    norm_num[differentiableAt_of_deriv_ne_zero (mt (.▸this M) _),id]
  field_simp only [ R, X, false_iff_true, AffineMap.lineMap_apply,deriv]at*
  refine fderiv.comp M (h₁.differentiable ↑le_rfl _) ((differentiableAt_id'.smul_const _).add_const _)▸(? _)
  suffices:a-B=∑b,(a b-B b) •1 •Pi.single b 1
  · norm_num[this▸map_sum _ _ _,deriv_add_const _,deriv_smul_const]
    by_cases h: a ↑0 =B 0
    · norm_num[mul_right_comm, mul_self_pos.2 (x ∘ _), Fin.forall_fin_two, sub_eq_zero,funext_iff,h₂,h]
    specialize h₃$ M •(a-B)+B
    simp_rw [ (by ·norm_num [funext_iff, Fin.forall_fin_two]:![0,01]= Pi.single @1 (1:Real) ∧![(1 : ℝ),0]=Pi.single @0 1)]
      ↪ at h₃
    specialize h₂ (M • (a-B)+B) 1 1
    revert h₂ h₃ h
    generalize T:(fderiv ℝ f _)=m
    use (λ (i R L) =>by nlinarith[sq (m (Pi.single 1 1) (1)*2*(a (1)-B (1))+(m (Pi.single 0 1) (1) + m ↑(Pi.single 01 1)
      ↪ (0))*(a 0-B 0)),sq_pos_of_ne_zero $ sub_ne_zero.mpr i])
  exact (funext (by norm_num[Pi.single_apply, abs]))
```

Supplementary Figure 6: A proof found by AlphaProof for an analysis problem involving partial derivatives. This is `putnam_2018_b5` from PutnamBench.
